# Mutations in the pantothenate kinase of *Plasmodium falciparum* confer diverse sensitivity profiles to antiplasmodial pantothenate analogues

**DOI:** 10.1371/journal.ppat.1006918

**Published:** 2018-04-03

**Authors:** Erick T. Tjhin, Christina Spry, Alan L. Sewell, Annabelle Hoegl, Leanne Barnard, Anna E. Sexton, Ghizal Siddiqui, Vanessa M. Howieson, Alexander G. Maier, Darren J. Creek, Erick Strauss, Rodolfo Marquez, Karine Auclair, Kevin J. Saliba

**Affiliations:** 1 Research School of Biology, The Australian National University, Canberra, Australia; 2 Department of Chemistry, University of Glasgow, Glasgow, United Kingdom; 3 Department of Chemistry, McGill University, Montreal, Quebec, Canada; 4 Department of Biochemistry, Stellenbosch University, Matieland, South Africa; 5 Monash Institute of Pharmaceutical Sciences, Monash University, Melbourne, Australia; 6 Department of Chemistry, Xi’an Jiaotong-Liverpool University, Suzhou, China; 7 Medical School, The Australian National University, Canberra, Australia; U Tex SouthWestern, UNITED STATES

## Abstract

The malaria-causing blood stage of *Plasmodium falciparum* requires extracellular pantothenate for proliferation. The parasite converts pantothenate into coenzyme A (CoA) via five enzymes, the first being a pantothenate kinase (*Pf*PanK). Multiple antiplasmodial pantothenate analogues, including pantothenol and CJ-15,801, kill the parasite by targeting CoA biosynthesis/utilisation. Their mechanism of action, however, remains unknown. Here, we show that parasites pressured with pantothenol or CJ-15,801 become resistant to these analogues. Whole-genome sequencing revealed mutations in one of two putative PanK genes (*Pfpank1*) in each resistant line. These mutations significantly alter *Pf*PanK activity, with two conferring a fitness cost, consistent with *Pfpank1* coding for a functional PanK that is essential for normal growth. The mutants exhibit a different sensitivity profile to recently-described, potent, antiplasmodial pantothenate analogues, with one line being *hypersensitive*. We provide evidence consistent with different pantothenate analogue classes having different mechanisms of action: some inhibit CoA biosynthesis while others inhibit CoA-utilising enzymes.

## Author summary

The coenzyme A (CoA) biosynthetic pathway is under investigation as a target for the development of drugs aimed at several infectious agents, including malaria parasites. To synthesise CoA, the parasite scavenges the essential precursor pantothenate (vitamin B_5_). Several pantothenate analogues possess potent (nM) activity against the parasite, but their exact mechanism of action is unknown. We have generated mutant parasites that are resistant or hypersensitive to various pantothenate analogues. These parasites harbour mutations in a gene we now show codes for the first enzyme in the CoA biosynthesis pathway. This enzyme is not the target of the analogues, but instead converts them into antimetabolites that, depending on the analogue, either inhibit a CoA biosynthesis enzyme or downstream CoA-utilising enzymes.

## Introduction

In recent years, the effort to roll back malaria has shown encouraging progress through the increased use of insecticide-treated mosquito nets, improved diagnostics and artemisinin-based combination chemotherapies (ACTs) [1]. Evidence of this includes the decreasing worldwide malaria incidence (266 million cases in 2005 down to 212 million cases in 2015) and mortality (741,000 deaths in 2005 down to 429,000 deaths in 2015) over the past decade [1]. However, there is an alarming trend of ACT-resistant parasites emerging in multiple Asian countries where the disease is endemic [2]. Recently, there have also been multiple reports of patients contracting ACT-resistant *Plasmodium falciparum* malaria from various African countries [3,4], which exemplify the clear risk of artemisinin resistance developing in the continent. This threat to the efficacy of ACTs highlights the requirement for a new armoury of antimalarial medicines, with several compounds representing different chemotypes entering the preclinical trial stage. However, the antimalarial drug-discovery pipeline is reliant on just a few known drug targets and the probability of successfully producing a new blood-stage medicine remains low [5]. In order to manage the threat of parasite drug resistance, there needs to be a continued effort to identify new classes of antimalarials. One metabolic pathway that has garnered recent interest for drug-development is the parasite’s coenzyme A (CoA) biosynthetic pathway [6,7].

**Fig 1:**
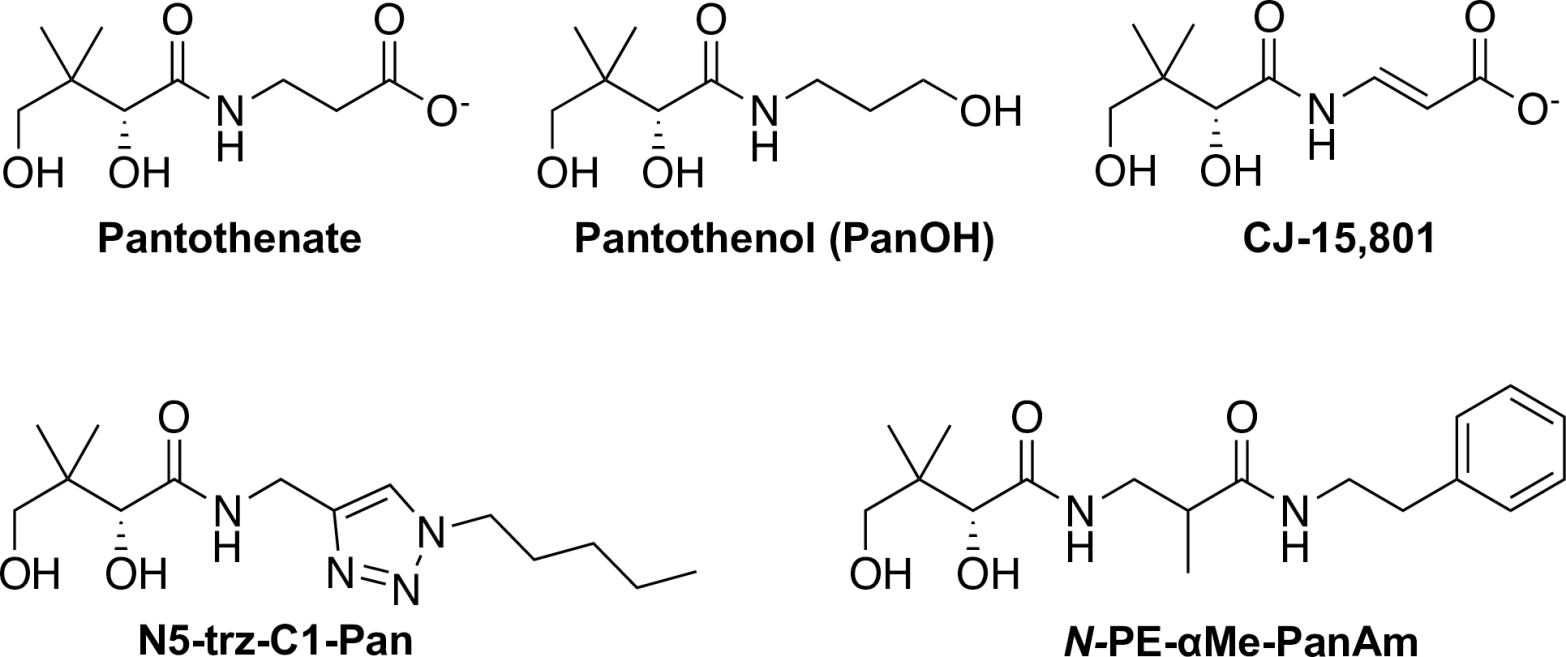
Structures of pantothenate and the pantothenate analogues used in this study.

Early seminal studies have shown that the asexual stage of intra-erythrocytic *P*. *falciparum* absolutely requires an exogenous supply of vitamin B_5_ (pantothenate; **Fig 1**) for survival [7–9]. Pantothenate is taken up by the parasite [10,11] and converted into CoA, an essential cofactor for many metabolic processes [7]. This conversion is catalysed by a series of five enzymes, the first of which is pantothenate kinase (*Pf*PanK), an enzyme that phosphorylates pantothenate to form 4’-phosphopantothenate [11]. By performing this step, the parasite traps pantothenate within its cytosol and commits it to the CoA biosynthetic pathway [10]. The additional four steps are, in turn, catalysed by phosphopantothenoylcysteine synthetase (*Pf*PPCS), phosphopantothenoylcysteine decarboxylase (*Pf*PPCDC), phosphopantetheine adenylyltransferase (*Pf*PPAT) and dephospho-CoA kinase (*Pf*DPCK) [6]. Putative genes coding for each of the enzymes in the pathway (with several enzymes having multiple putative candidates) have been identified in the *P*. *falciparum* genome [12,13] and have also been shown to be transcribed during the intraerythrocytic stage of the parasite’s lifecycle [14]. In order to capitalise on the pathway as a potential target for drug-discovery, however, it is crucial to ascertain the exact identity of each of these putative genes. This will allow the process to become more efficient and targeted.

Investigations aimed at discovering antiplasmodial agents that act by interfering with the parasite’s CoA biosynthetic pathway identified several antiplasmodial pantothenate analogues, including pantothenol (PanOH) and CJ-15,801 [9,15,16] (see **Fig 1** for structures). Subsequent studies identified pantothenamides as antiplasmodial pantothenate analogues with substantially increased potency [17–19]. Unfortunately pantothenamides are unstable *in vivo* because they are degraded by the serum enzyme pantetheinase [17]. Recent reports of structural optimisations of lead pantothenamides have described two compounds, N5-trz-C1-Pan (compound 1e in Howieson *et al*. [20]) and *N*-PE-αMe-PanAm (see **Fig 1** for structures), that are potent antiplasmodials (with nanomolar IC_50_ values) and also resistant to pantetheinase-mediated degradation [20,21]. However, although these compounds have been shown to target CoA biosynthesis or utilisation, their exact mechanism(s) of action has not been elucidated.

In this study, we have used continuous drug-pressuring with PanOH or CJ-15,801 to generate a number of *P*. *falciparum* parasite lines that are several-fold resistant to these pantothenate analogues. Whole-genome sequencing revealed mutations in one of the two putative *Pfpank* genes of all of the clones, *Pfpank1*. Complementation experiments confirmed that these mutations are responsible for the resistance phenotypes. Characterisation of the effects of the mutations on parasite growth in culture and also *Pf*PanK function, generated data consistent with the mutated gene coding for an active PanK in *P*. *falciparum* and with the gene being essential for normal parasite development during the intraerythrocytic stage of its lifecycle. Additional characterisation of the PanOH and CJ-15,801-resistant lines revealed that antiplasmodial pantothenate analogues have at least two distinct mechanisms of action, targeting CoA biosynthesis or utilisation. Both of these mechanisms can be influenced by the *Pfpank1* mutations identified here. Furthermore, our study provides genetic evidence validating the importance of the metabolic activation of pantothenate analogues in the antiplasmodial activity of these compounds.

## Materials and methods

### Parasite culture and lysate preparation

*P*. *falciparum* parasites were maintained in RPMI 1640 media supplemented with 11 mM glucose (final concentration of 22 mM), 200 *μ*M hypoxanthine, 24 *μ*g/mL gentamicin and 6 g/L Albumax II (referred to as complete medium) as previously described [22]. Clonal parasite populations were generated through limiting dilution cloning as reported previously [23], with modifications. Parasite lysates were prepared from saponin-isolated mature trophozoite-stage parasites as described previously [10].

### Plasmid preparation and parasite transfection

Several plasmid constructs were generated through the course of this study to be used for different lines of investigations. The strategies used to generate the *Pfpank1*-pGlux-1, *Pfpank1*-stop-pGlux-1 and Δ*Pfpank1*-pCC-1 plasmids are detailed in the **SI** and the primers used in these strategies are listed in **S1 Table**. The constructs were transfected into ring-stage parasites and positive transfectants were selected by introducing WR99210 (10 nM) [24].

### Compound synthesis

The pantothenate analogues CJ-15,801 [25], N5-trz-C1-Pan [26] and *N*-PE-αMe-PanAm [21], used in this study were synthesised as reported previously.

### SYBR Safe-based parasite proliferation assay

The effect of various compounds on the *in vitro* proliferation of the different parasite lines were tested using a previously-reported SYBR Safe-based fluorescence assay [17], with minor modifications (**SI**). *In vitro* pantothenate requirement experiments were performed similarly (**SI**), except instead of a test compound, ring stage-infected erythrocytes were incubated in pantothenate-free complete RPMI 1640 medium (made complete as described above; Athena Enzyme Systems) supplemented with 2-fold serial dilutions of pantothenate. IC_50_ and SC_50_ (defined in the Results section) values were determined from the sigmoidal curves fitted to each data set using nonlinear least squares regression (**SI**).

### Drug pressuring

Two independent drug-pressuring cultures were initiated for each of the pantothenate analogues, PanOH and CJ-15,801. Pressuring was initiated by exposing synchronous ring-stage Parent line parasites (10 mL culture of 2 or 4% parasitaemia and 2% haematocrit) to either analogue at the IC_50_ values obtained for the Parent line at the time (PanOH = 400 *μ*M and CJ-15,801 = 75 *μ*M). Parasites were then exposed to cycles of increasing drug-pressure that lasted about 2–4 weeks each (**SI**). When the pressured parasites became approximately 8 × less sensitive than the Parent line to the selecting analogues, they were cloned and cultured in the absence of the analogues for the remainder of the study.

### Competition assay

In order to compare the fitness of the mutant clones with that of the Parent, we set up three competition cultures, each containing a mixture of one mutant line and the Parent line. Equal number of parasites (5 × 10^8^ cells in the first experiment and 2.5 × 10^8^ cells in the second experiment) from each line were mixed into a single culture. Aliquots (3 to 5 mL) of these cultures were immediately used for a PanOH SYBR Safe-based parasite proliferation assay (to generate Week 0 data) as described above. The cultures were then maintained under standard conditions as detailed above for a period of 6 weeks before they were used to perform another PanOH proliferation assay (to generate Week 6 data).

### Whole genome sequencing and variant calling

Next generation whole genome sequencing was performed by the Biomolecular Resource Facility at the Australian Cancer Research Foundation, the Australian National University. Samples were sequenced with the Illumina MiSeq platform with version 2 chemistry (2 × 250 base pairs, paired-end reads) Nextera XT Kit (Illumina). To determine the presence of any single nucleotide polymorphisms (SNPs) in the genomes of the drug-pressured clones, the genomic sequencing data were analysed using an integrated variant calling pipeline, PlaTyPus, as previously described [27], with minor modifications to resolve operating system incompatibility. As PlaTyPus does not detect insertions-deletions polymorphisms (“indels”), the Integrated Genome Viewer (IGV) software (Broad Institute) was used to manually inspect the gene sequences of all putative enzymes in the CoA biosynthetic pathway for indels.

### Quantitative polymerase chain reaction (qPCR)

To determine the proportions of parasites (i.e. Parent versus mutant clone) that make up each competition culture, we performed qPCR to measure the amount of Parent and mutant genomic DNA (gDNA) at week 0 and week 6 of the competition assay. gDNA was extracted using a DNeasy Plant Mini Kit (QIAGEN) or QIAamp DNA Blood Midi Kit (QIAGEN) following the manufacturer’s instructions, unless otherwise specified (detailed in **SI**). Six different primer sets were designed, each to detect the specific variants (SNPs or indel) in the wild-type and mutant *Pfpank1* alleles (**S1 Table**). The qPCRs were performed using QuantiTect 2 × SYBR Green PCR Master Mix (QIAGEN) essentially as reported previously [28] with the following specifications: The reactions (20 *μ*L final volume) for “Parent vs PanOH-A” and “Parent vs PanOH-B” contained 2 *μ*L of 10 ng/*μ*L gDNA stocks while that for “Parent vs CJ-A” contained 4 *μ*L of 10 ng/*μ*L gDNA stocks. The PCR program for “Parent vs PanOH-A” and “Parent vs CJ-A” entailed an initial DNA polymerase activation step (95°C for 15 min) followed by 40 cycles of denaturation (94°C for 15 s), annealing (60 or 55°C, respectively, for 30 s) and extension (72°C for 30 s) with the detection step (green channel) set during the extension step of each cycle. For “Parent vs PanOH-B” the PCR was programmed as above except that it was set for 45 cycles, the annealing step was carried out at 58°C and included an additional detection step (65°C for 15 s, green channel) in each cycle. Following each reaction, a melt curve analysis was performed starting with a temperature that is 1°C lower than the annealing temperature and increased to 99°C at 1°C/s. Control reactions that each contained defined proportions of the Parent and mutant gDNA as templates, set at the same final DNA concentration as detailed above, were included in each qPCR experiment to enable preparation of a standard curve. The standard curve was generated by plotting the threshold cycle values obtained for the control reactions against the corresponding log_10_ gDNA amount and fitting a straight line (*y* = *y*0 +*ax*, where *y* denotes threshold cycle value, *x* denotes log_10_ DNA amount and *a* denotes primer efficiency). The concentration of Parent and mutant gDNA in each sample was determined by comparing their threshold cycle against the appropriate standard curve. All reactions were performed in duplicate.

### Generation of *Pf*PanK1 model

The structure of *Pf*PanK1 minus its parasite-specific inserts was predicted by homology modeling using the AMPPNP and pantothenate-bound human PanK3 structure (PDB ID: 5KPR [29]) as a template. *Pf*PanK1 shares 28% sequence identity with human PanK3 over the parts of the protein that have been modeled. The model was generated using the one-to-one threading module of the Phyre2 webserver (available at http://www.sbg.bio.ic.ac.uk/phyre2) [30].

### Confocal microscopy

Erythrocytes infected with trophozoite-stage 3D7 strain *P*. *falciparum* parasites expressing *Pf*PanK1-GFP were observed and imaged either with a Leica TCS-SP2-UV confocal microscope (Leica Microsystems) using a 63 × water immersion lens or a Leica TCS-SP5-UV confocal microscope (Leica Microsystems) using a 63 × oil immersion lens. The parasites were imaged as fixed or live cells as described in the **SI**.

### Attempted disruption of *Pfpank1*

The *Pfpank1* disruption plasmid, Δ*Pfpank1*-pCC-1 (**SI**), was transfected into wild-type 3D7 strain *P*. *falciparum*, and positive transfectants were selected as described above. *P*. *falciparum* parasites have previously been shown to survive equally well in a pantothenate-free complete RPMI 1640 medium supplemented with ≥100 *μ*M CoA as compared to standard complete medium, consistent with them having the capacity to take up exogenous CoA, hence bypassing the need for any *Pf*PanK activity [7]. Therefore, to support the growth of any *Pfpank1* gene-disrupted parasites generated with the Δ*Pfpank1*-pCC-1 construct, parasites were continuously maintained in complete medium supplemented with 100 *μ*M CoA following transfection. Positive and negative selection steps (with WR99210 and 5-fluorocytosine (5-FC), respectively) were performed to isolate Δ*Pfpank1*-pCC-1-transfectant parasites in which the double crossover homologous recombination had occurred (detailed in **SI**).

### Southern blot analysis

gDNA samples (~2 *μ*g) extracted from Δ*Pfpank1*-pCC-1-transfectant parasites isolated through the positive and negative selection steps were digested with the restriction enzyme *Afl*II (New England Biolabs), before being analysed by Southern blotting using the digoxigenin (DIG) system (Roche) according to the Roche DIG Applications Manual for Filter Hybridisation. The results from the Southern blot were confirmed with PCR (the primers used are shown in **S1 Table**).

### [^14^C]Pantothenate phosphorylation by parasite lysate

The phosphorylation of [^14^C]pantothenate by parasite lysates prepared from the Parent and mutant clonal lines was measured using Somogyi reagent (which precipitates phosphorylated compounds from solution) as outlined previously [31], with some modifications (detailed in **SI**).

### Metabolism of N5-trz-C1-Pan

Cultures of predominantly trophozoite-stage *P*. *falciparum*-infected erythrocytes (Parent line) were concentrated to >95% parasitaemia using magnet-activated cell sorting as described elsewhere [32]. Following recovery, trophozoite-infected erythrocytes were treated with N5-trz-C1-Pan (1 *μ*M) or a solvent control (0.01% v/v DMSO) before the metabolites in these samples were extracted and processed for liquid chromatography-mass spectrometry (LC-MS) analysis. Metabolite samples were analysed by LC-MS, using a Dionex RSLC U3000 LC system (ThermoFisher) coupled with a high-resolution, Q-Exactive MS (ThermoFisher), as described previously [33] (detailed in **SI**). LC-MS data were analysed in a non-targeted fashion using the IDEOM workflow, as described elsewhere [34]. Unique features identified in N5-trz-C1-Pan-treated samples were manually assessed by visualising high resolution accurate mass LC-MS data with Xcalibur Quanbrowser (ThermoFisher) software.

### *Pf*PanK1 protein levels

To measure *Pf*PanK1 levels, parasite samples were prepared from Parent and mutant cultures as previously described with minor modifications [35]. Briefly, mature trophozoites were saponin-isolated from a culture of infected erythrocytes (10% parasitaemia, 2% haematocrit in 30 mL) by resuspending the pellet in 1 × phosphate buffered saline (PBS; Sigma-Aldrich) containing 0.05% (w/v) saponin, 1 × complete mini protease inhibitor cocktail (Roche), 20 mM sodium fluoride and 0.1 mM sodium orthovanadate. The isolated trophozoites were pelleted (2,000 × *g*, 8 min), washed (15,850 × *g*, 30 s, supernatant discarded after each wash) once in 1 mL of the above solution excluding saponin, and twice with 1 mL 1 × PBS. The trophozoite pellet was then stored at −80°C until required. For sample processing, a total of 500 *μ*g of protein, accurately determined using the Pierce BCA protein assay kit (ThermoFisher), was incubated overnight with sequencing-grade trypsin (1:50 dilution; Promega). On the following day, trypsin activity was quenched using 5% formic acid (FA) and the detergent (sodium deoxycholate) used for protein solubilisation was removed. The samples were then dried and resuspended in 20 *μ*L of 2% (v/v) acetonitrile (ACN) and 0.1% (v/v) FA for LC-MS/MS analysis. For facilitating retention-time (RT) alignments among samples, a RT kit [36] (iRT kit, Biognosys) was spiked into all samples (1:20 dilution).

LC-MS/MS was carried out as described previously [35], with minor modifications. Briefly, samples were loaded at a flow rate of 15 *μ*L/min onto a reversed-phase trap column (100 *μ*m × 2 cm; Acclaim PepMap media, Dionex), which was maintained at a temperature of 40°C. Peptides were then eluted from the trap column at a flow rate of 0.25 *μ*L/min through a reversed-phase capillary column (75 *μ*m × 50 cm; LC Packings, Dionex). The HPLC gradient was set to 158 min using a gradient that reached 30% ACN after 123 min, then 34% ACN after 126 min, 79.2% ACN after 131 min and 2% ACN after 138 min, at which it was maintained for a further 20 min. The mass spectrometer was operated in a data-independent acquisition (DIA) mode with a 25-fixed-window setup of 24 *m/z* effective precursor isolation over the *m/z* range of 375–975 Da.

For Spectronaut processing, raw files were loaded into Spectronaut (version 11, Biognosys) with an in-house *P*. *falciparum*-infected red blood cell library and default settings. Briefly, RT prediction type was set to dynamic iRT and the correction factor for the window was set to one. Mass calibration was set to local mass calibration. Interference correction was on MS2 level. The false discovery rate was set to 1% at peptide precursor level. For quantification, the interference correction was activated and a cross run normalisation was performed using the total peak area as the normalisation base. A significance filter of 0.01 was used. The peptides used in the identification and quantitation of *Pf*PanK1 and the four control proteins are listed in **S2 Table**.

### Statistical analysis

Statistical analysis of means was carried out with unpaired, two-tailed, Student’s *t* tests using GraphPad 6 (GraphPad Software, Inc) from which the 95% confidence interval of the difference between the means (95% CI) was obtained. All regression analysis was done using SigmaPlot version 11.0 for Windows (Systat Software, Inc).

## Results

### *Pfpank1* mutations mediate parasite resistance to PanOH and CJ-15,801

**Fig 2:**
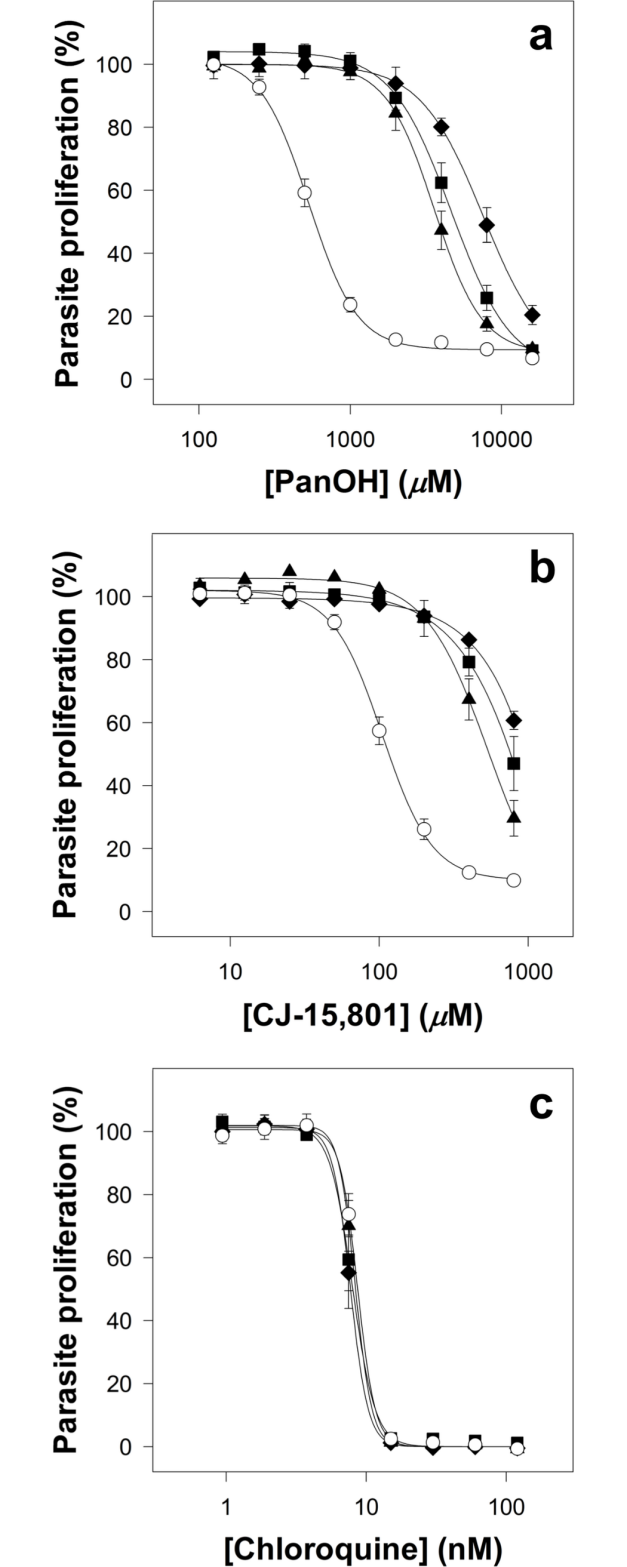
**Percentage proliferation of parasites from the Parent (white circles), PanOH-A (black triangles), PanOH-B (black squares) and CJ-A (black diamonds) lines in the presence of (a) PanOH, (b) CJ-15,801 or (c) chloroquine.** Drug-pressured lines were generated by exposing Parent line parasites to 11 − 13 weeks of continuous drug-pressuring with either PanOH (for PanOH-A and PanOH-B) or CJ-15,801 (for CJ-A), followed by limiting dilution cloning. Values are averaged from ≥ 4 independent experiments, each carried out in triplicate. All error bars represent SEM. Error bars are not visible if smaller than the symbols.

The 3D7 *P*. *falciparum* strain was cloned through limiting dilution, and a single parasite line (henceforth referred to as the Parent line) was used to generate all of the subsequent lines tested in this study (unless otherwise specified). This was done to ensure that all of the parasite lines generated during the course of this study would share a common genetic background. Using the Parent line, three independent drug-pressuring cultures were set up (two with PanOH and one with CJ-15,801). When these parasites had attained approximately 8-fold decrease in sensitivity (~11–13 weeks of continuous pressuring), they were subsequently cloned by limiting dilution and maintained in the absence of drug pressure. In this manner, three parasite clones were generated: PanOH-A and PanOH-B were generated from the two independent PanOH-pressured cultures while CJ-A was generated from the CJ-15,801-pressured culture. The clones are significantly resistant (95% confidence interval (CI) exclude 0) to the pantothenate analogues they were pressured with. The 50% inhibitory concentration (IC_50_) values of PanOH against PanOH-A and PanOH-B, and the IC_50_ value of CJ-15,801 against CJ-A are approximately 7–8-fold higher than those measured against the Parent line (**Fig 2A and 2B** and **S3 Table**). Significant cross-resistance towards the other pressuring analogue was observed for these clones, as compared to the Parent line (95% CI exclude 0). The PanOH-A and PanOH-B lines were found to be 4–6-fold less sensitive to CJ-15,801 while CJ-A was found to be 13-fold less sensitive to PanOH (**Fig 2A and 2B** and **S3 Table**). To ensure that the clones did not develop a general drug-resistance phenotype during the selection process, we tested their sensitivity to chloroquine, an antiplasmodial with a mechanism of action that is unrelated to the parasite’s CoA biosynthetic pathway [37]. We found that all of the drug-pressured lines have chloroquine IC_50_ values that are indistinguishable from that of the Parent line (95% CI include 0; **Fig 2C** and **S3 Table**).

**Fig 3:**
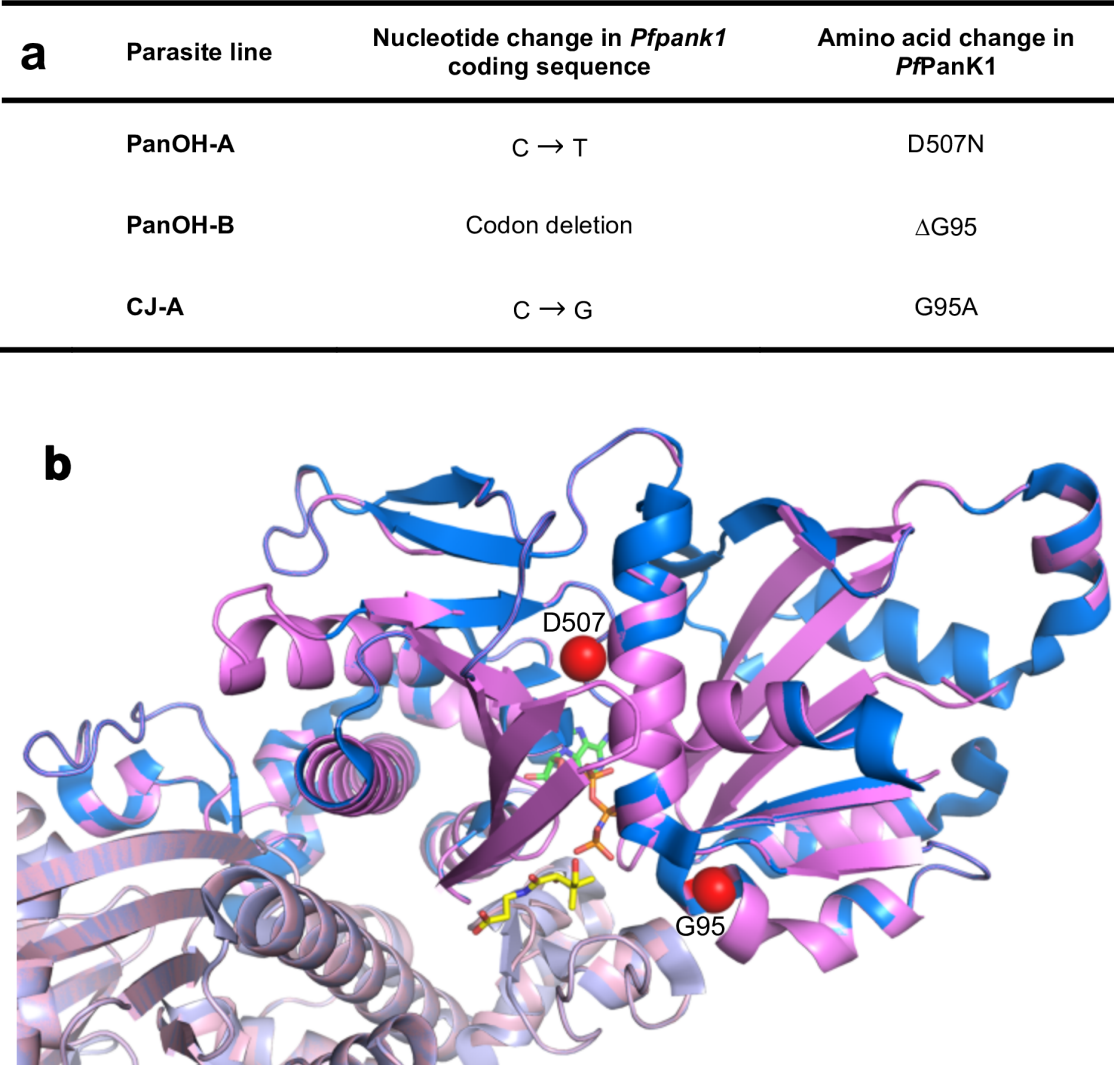
Mutations in *Pfpank1* and the affected residues within the *Pf*PanK1 protein. (a) The single nucleotide polymorphisms detected in the *Pfpank1* gene (accession number: PF3D7_1420600) of PanOH-A, PanOH-B and CJ-A, and the corresponding amino acid changes in the *Pf*PanK1 protein. (b) A three-dimensional homology model of the *Pf*PanK1 protein (pink) based on the solved structure of human PanK3 (PDB ID: 5KPR), overlaid on the human PanK3 structure in its active conformation (blue), with an ATP analogue (AMPPNP; carbon atoms coloured green) and pantothenate (carbon atoms coloured yellow) bound. *Pf*PanK1 shares 28% sequence identity with human PanK3 over the parts of the protein that have been modeled. Red spheres indicate the residues (G95 and D507) affected by the mutations in the parasite proteins. Human PanK3 has been shown to exist as a dimer. Here, individual monomers are shown in different shades of pink and blue.

The PanOH and CJ-15,801 resistance phenotypes observed in the clones were stable for several months of continuous culture in the absence of the pressuring analogue (≥ 3 months), consistent with a genetic alteration in these parasites. To determine the mutation(s) responsible for these phenotypes, gDNA was extracted from each clone and subjected to whole genome sequencing. All of the drug-resistant clones were found to harbour a unique mutation in the putative pantothenate kinase gene, *Pfpank1* (PF3D7_1420600), as shown in **Fig 3A**. Other non-synonymous mutations were detected for each clone (**S4 Table**) but we did not find another gene that is mutated in all three clones. The mutation found in the *Pfpank1* of PanOH-A results in the substitution of Asp507 for Asn. The other two drug-resistant clones have a mutation at position 95 of the protein: the *Pfpank1* of PanOH-B harbours a deletion of an entire codon leading to a loss of Gly from the *Pf*PanK1 protein, while the *Pf*PanK1 of CJ-A has a Gly to Ala substitution. Since the structure of *Pf*PanK1 has not yet been resolved, we generated a three-dimensional model in order to map the mutations within the enzyme. **Fig 3B** shows a *Pf*PanK1 model structure (pink) based on the solved structure of human PanK3 in complex with adenylyl-imidodiphosphate (AMPPNP) and pantothenate (PDB ID: 5KPR), overlaid on this structure (blue). The spheres shown in the model indicate the positions of the mutated residues, while the bound AMPPNP and pantothenate indicate the active site of the enzymes. Although the mutations are far apart in the primary amino acid sequence of *Pf*PanK1, they are positioned in closer proximity to each other in the folded protein and are situated adjacent to the active site.

**Fig 4:**
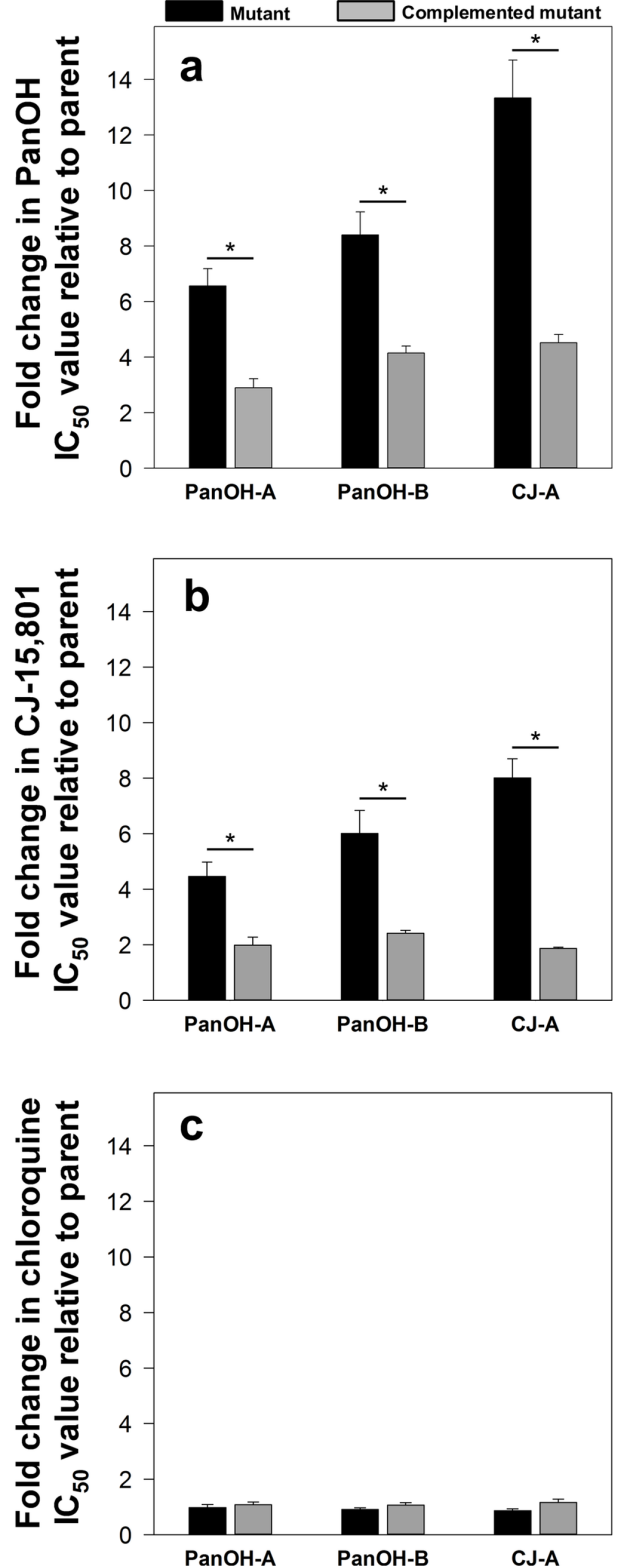
**The fold change in (a) PanOH, (b) CJ-15,801 and (c) chloroquine IC_50_ values of PanOH-A, PanOH-B and CJ-A parasite lines, in the absence (black bars) or presence (grey bars) of wild-type *Pf*PanK1 complementation, relative to that of their corresponding parental lines.** Each fold change value is averaged from ≥ 3 independent experiments and error bars represent SEM. An asterisk indicates that the fold change in sensitivity of the mutant is significantly altered by complementation (PanOH fold change 95% CI: PanOH-A = -5.29 to -2.05, PanOH-B = -6.25 to -2.26 & CJ-A = -13.00 to -4.62; CJ-15,801 fold change 95% CI: PanOH-A = -4.17 to -0.79, PanOH-B = -6.14 to -1.06 & CJ-A = -8.05 to -4.22). No change in chloroquine sensitivity was observed (95% CI: PanOH-A = -0.23 to 0.43, PanOH-B = -0.09 to 0.40 & CJ-A = -0.01 to 0.59). The fold change in the sensitivity to CJ-15,801 for PanOH-B and CJ-A before and after complementation was calculated using IC_35_ values.

To confirm that the resistance phenotypes observed for the clones are directly caused by the mutations in *Pfpank1*, each clone was transfected with an episomal plasmid (*Pfpank1*-stop-pGlux-1) that enables the parasites to express the wild-type copy of *Pfpank1* (in addition to the endogenous mutated copy). These complemented lines are indicated with a superscripted “+WT*Pf*PanK1”. From **Fig 4** (and **S3 Table**), it can be observed that the complemented mutant clones (grey bars) are significantly less resistant to PanOH (**Fig 4A**) and CJ-15,801 (**Fig 4B**) compared to the non-complemented mutant clones (black bars; 95% CI exclude 0). As expected, the relative sensitivity of the mutant clones to chloroquine is unchanged by the presence of the *Pf*PanK1-encoding plasmid (95% CI include 0; **Fig 4C**). Transfection of the *Pf*PanK1-encoding plasmid into the Parent line did not alter its sensitivity to PanOH, CJ-15,801 or chloroquine (95% CI include 0; **S3 Table**). These data are consistent with the mutations observed in *Pfpank1* being responsible for the resistance phenotype observed in the mutant clones. An alternative strategy to confirm that the mutant *Pf*PanK1 is responsible for the observed phenotypes would be to express the mutant forms of *Pf*PanK1 in the Parent line. This has not, however, been attempted.

### *Pfpank1* mutations impair parasite proliferation

**Fig 5:**
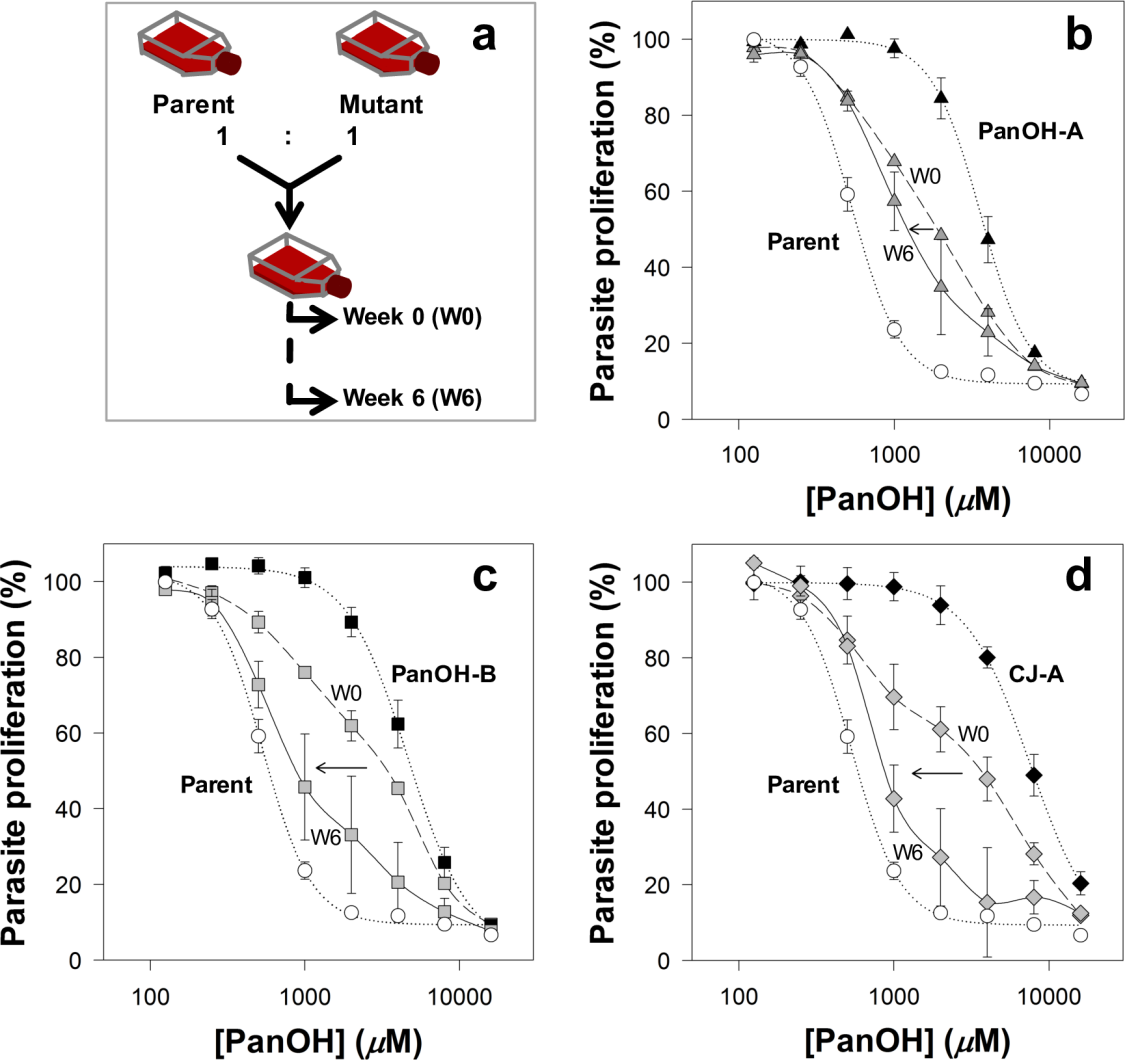
The fitness of the different mutant lines generated in this study relative to the Parent line as determined from parasite competition assays. (a) A flow chart illustrating how the competition assay was performed. For each competition culture, an equal number of parasites from the Parent and a mutant line were combined into a single flask. These mixed cultures were maintained for a period of 6 weeks. The fitness cost associated with the *Pfpank1* mutations was assessed by determining the PanOH sensitivity of the mixed cultures: (b) Parent vs PanOH-A (grey triangles), (c) Parent vs PanOH-B (grey squares) and (d) Parent vs CJ-A (grey diamonds), at Week 0 (W0; dashed lines) and Week 6 (W6; solid lines). It was expected that the greater the fitness cost to the mutant, the greater the shift of its mixed culture PanOH dose-response curve toward the Parent line curve after 6 weeks. Arrows indicate this shift between W0 and W6. The parasite proliferation curves (dotted lines) of the respective mutant clones (black symbols) and Parent line (white circles) are also shown for comparison. Values for the mixed cultures are averaged from 2 independent experiments, each carried out in triplicate. Error bars represent SEM (n ≥ 4) for the individual cultures and range/2 for the mixed cultures, and are not visible if smaller than the symbols.

We performed a competition assay to determine whether the *Pfpank1* mutations impart a fitness cost to the parasite clones. Each of the competition cultures was set up by mixing an equal number of parasites from the Parent line and one of the mutant clonal lines, and was maintained under standard conditions for a period of 6 weeks (**Fig 5A**). The sensitivity of each competition culture to PanOH was tested on the day the lines were mixed (Week 0) and again at the end of the 6-week period (Week 6). As expected, each Week 0 (dashed line) PanOH dose-response curve is between those obtained for the Parent and the respective mutant clone (dotted lines). A shift of the dose-response curve obtained at week 6 (solid line) towards the dose-response curve of the Parent line would indicate that the mutant *Pf*PanK1 imparts a fitness cost on the clone. As shown in **Fig 5B**, the Week 6 curve for the PanOH-A competition culture only exhibits a marginal leftward shift from Week 0, whereas those for PanOH-B (**Fig 5C**) and CJ-A (**Fig 5D**) exhibits a more substantial shift, almost reaching the dose-response curve of the Parent line (dotted line, white circles). These trends are supported by qPCR analysis designed to determine the proportions of mutant versus Parent gDNA at week 0 and week 6 (**S1 Fig**). These results are consistent with the mutations at position 95 in the *Pf*PanK1 of PanOH-B and CJ-A having a greater negative impact on the *in vitro* growth of the parasites. We also investigated the importance of *Pf*PanK1 expression for parasite growth by attempting to disrupt the *Pfpank1* locus in wild-type 3D7 parasites through homologous recombination (**S2A Fig**). However, using Southern blots, we failed to detect the presence of transfectants with the expected gene-knockout integration event (**S2B Fig**), consistent with this gene being essential during the parasite’s intraerythrocytic stage. The lack of integration of the knockout construct into the *Pf*PanK1 locus, even in a small sub-population of the parasite culture, was confirmed using PCR (**S2C Fig**).

### *Pf*PanK1 is a functional pantothenate kinase located within the parasite cytosol

**Fig 6:**
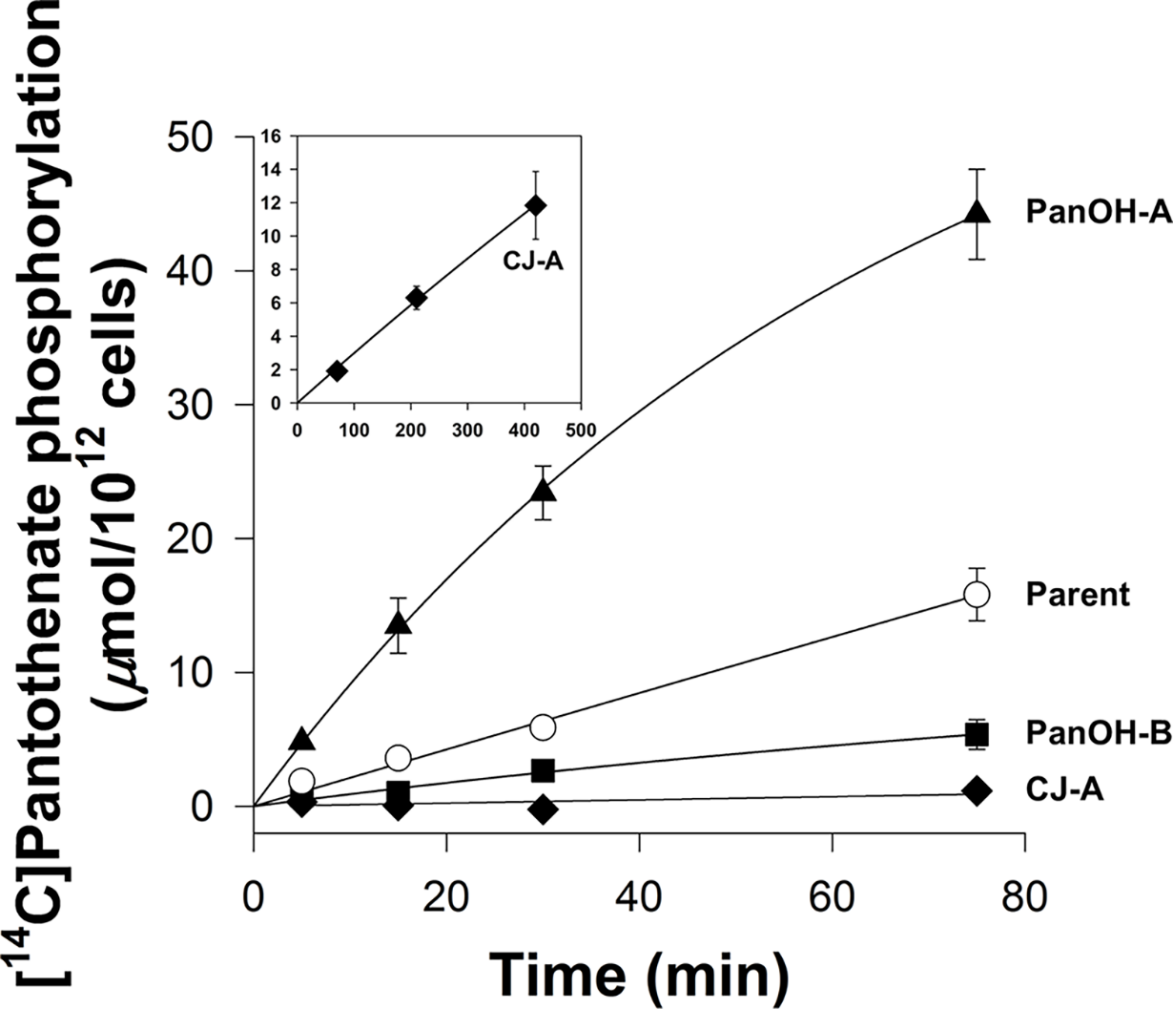
The phosphorylation of [^14^C]pantothenate (2 *μ*M) over time (in minutes) by lysates generated from Parent (white circles), PanOH-A (black triangles), PanOH-B (black squares) and CJ-A (black diamonds) parasites. Inset shows [^14^C]pantothenate (2 *μ*M supplemented with non-radioactive pantothenate to a total pantothenate concentration of 200 *μ*M) phosphorylation by CJ-A parasite lysates measured over 420 min. Values are averaged from 3 independent experiments, each performed with a different batch of lysate and carried out in duplicate. Error bars represent SEM and are not visible if smaller than the symbols.

**Fig 7:**
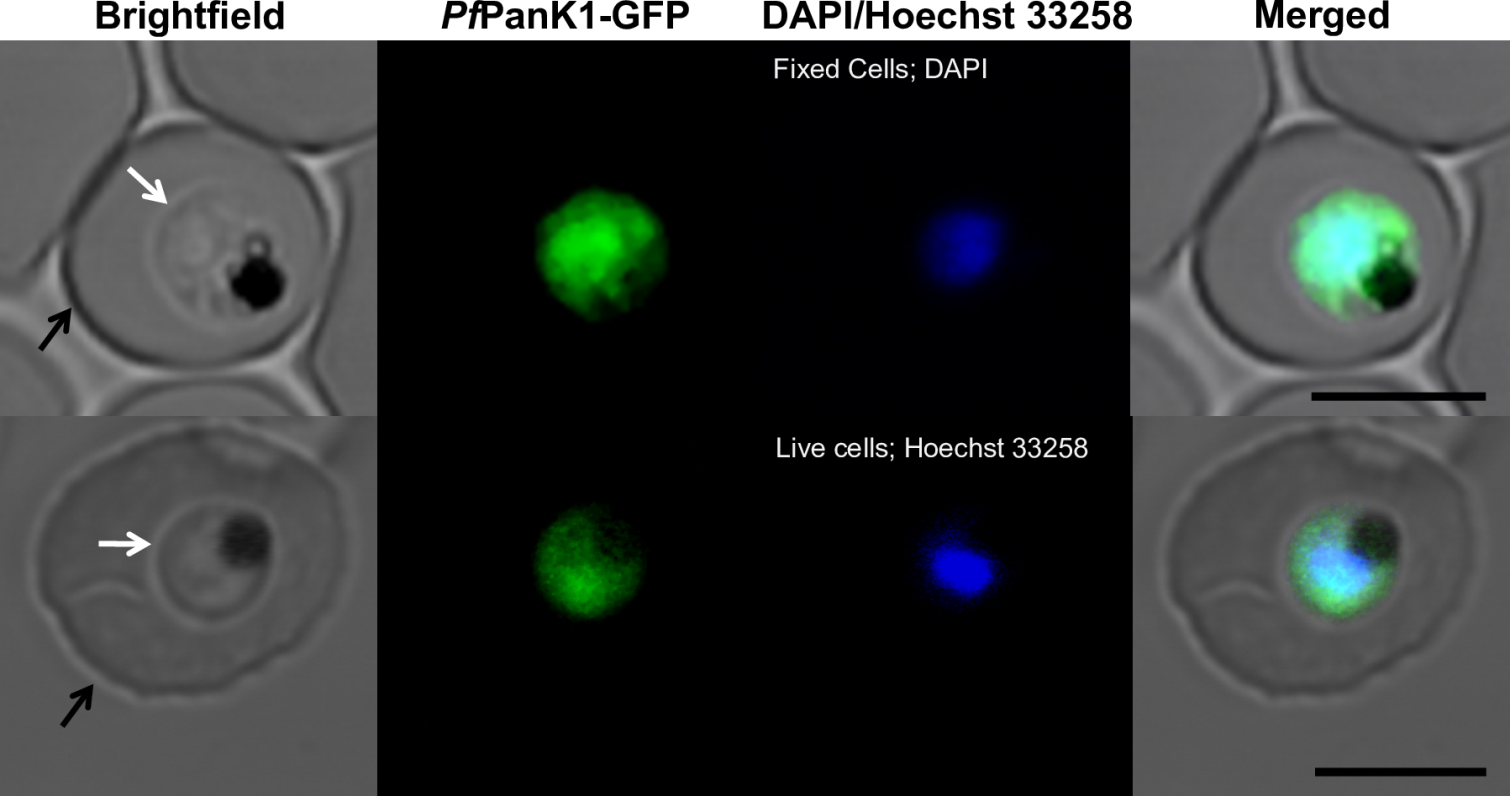
Confocal micrographs showing the subcellular location of GFP-tagged *Pf*PanK1 in 3D7 strain parasites harbouring the *Pfpank1*-pGlux-1 episomal plasmid. From left to right: Brightfield, GFP-fluorescence, DAPI- (fixed cells; top) or Hoechst 33258- (live cells; bottom) fluorescence, and merged images of erythrocytes infected with trophozoite-stage *P*. *falciparum* parasites expressing *Pf*PanK1-GFP. Arrows indicate the plasma membranes of either the erythrocytes (black) or the parasites (white). Scale bar represents 5 *μ*m.

The phosphorylation of radiolabelled pantothenate by lysates prepared from each of the mutant clones and the Parent line was measured to determine if the mutations in the putative *Pfpank1* gene affect PanK activity, and hence whether the gene codes for a functional PanK. As shown in **Fig 6**, at the end of the 75 min time-course, the lysate prepared from PanOH-A phosphorylated approximately 3 × more [^14^C]pantothenate than the lysate prepared from the Parent line, while the lysate of PanOH-B generated about 3 × less phosphorylated [^14^C]pantothenate compared to the Parent line. By comparison, the lysate prepared from CJ-A only produced a small amount of phosphorylated [^14^C]pantothenate in the same time period. The **inset** in **Fig 6** demonstrates that PanK activity could be detected in CJ-A lysates when the experiment was carried out in the presence of a 100-fold higher pantothenate concentration (200 *μ*M) and for an extended time (420 min). These observations provide strong evidence that the *Pfpank1* gene codes for a functional PanK. Further, we generated from the wild-type 3D7 strain a transgenic parasite line that episomally expresses a GFP-tagged copy of *Pf*PanK1 in order to localise the protein within the parasite. We found that *Pf*PanK1 is largely localised throughout the cytosol of trophozoite-stage parasites, and is not excluded from the nucleus (**Fig 7**).

**Fig 8:**
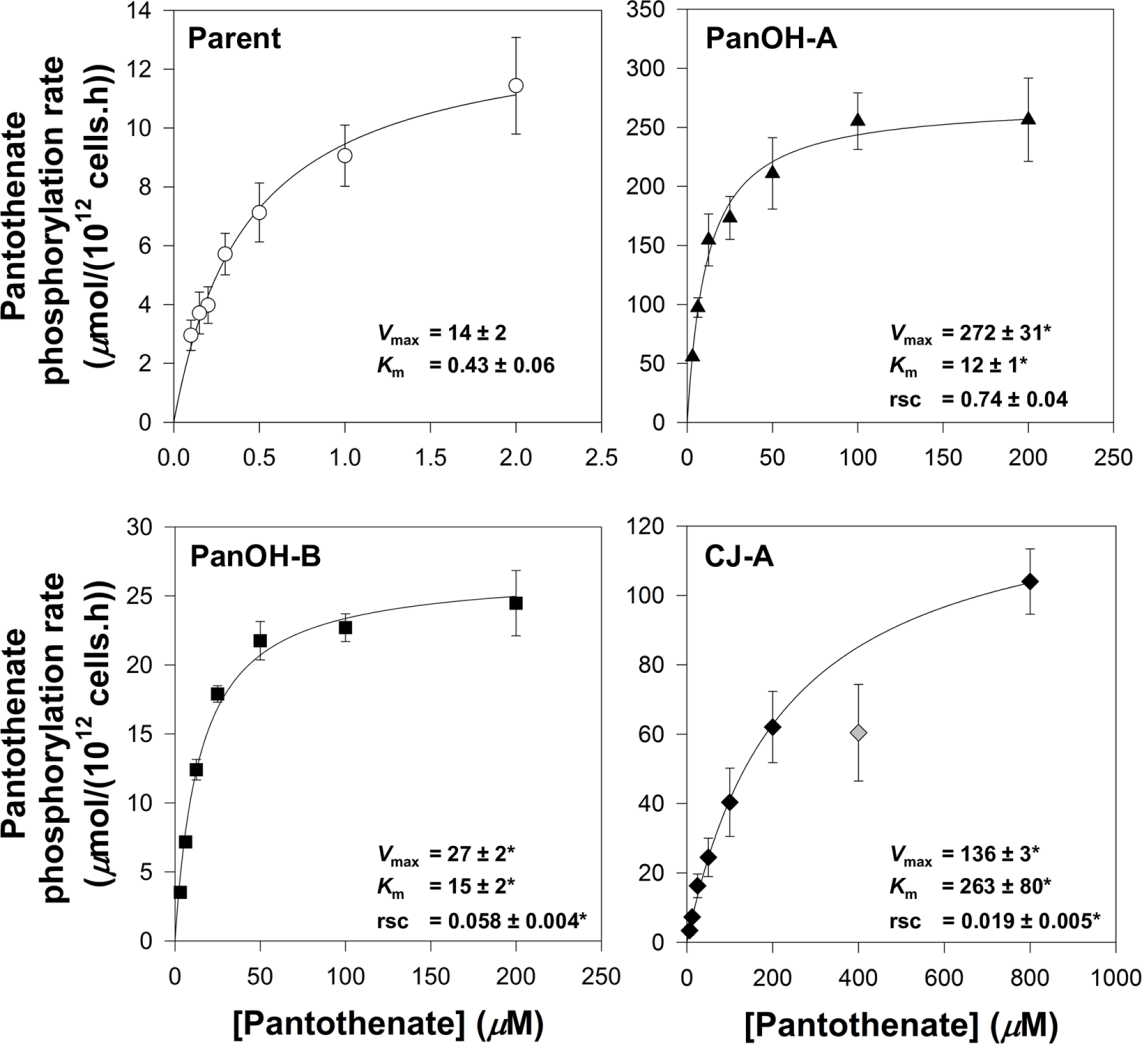
*Pf*PanK activity profiles derived from the initial rates of pantothenate phosphorylation by lysates generated from Parent (white circles), PanOH-A (black triangles), PanOH-B (black squares) and CJ-A (black diamonds) parasites at various pantothenate concentrations. The grey diamond indicates a data point that was outside the 95% confidence band when it was included in the non-linear regression. This data point was therefore deemed an outlier and was omitted from the data set used to generate the fitted curve shown. Values are averaged from 3 independent experiments, each performed with a different batch of lysate and carried out in duplicate. Error bars represent SEM and are not visible if smaller than the symbols. Relative specificity constant (rsc) values express the specificity constants obtained for each cell line relative to that of the Parent. The *V*_max_ (*μ*mol/(10^12^ cells.h)), *K*_m_ (*μ*M) and rsc values determined from the curves are shown within each box. Errors represent SEM (n = 3). An asterisk indicates that the value is significantly different compared to the Parent line (*V*_max_ 95% CI compared to Parent: PanOH-A = 173 to 344, PanOH-B = 5.2 to 21.6 & CJ-A = 112 to 133; *K*_m_ 95% CI compared to Parent: PanOH-A = 8.8 to 13.3, PanOH-B = 8.3 to 20.7 & CJ-A = 42 to 484; rsc 95% CI compared to Parent: PanOH-B = -1.366 to -0.519 & CJ-A = -1.404 to -0.557). No significant difference was observed between the rsc of the Parent and PanOH-A (95% CI compared to Parent: -0.698 to 0.179).

To investigate further the effects of the *Pf*PanK1 mutations present in the PanOH- and CJ-15,801-resistant clones, we analysed the PanK activity profiles using lysates prepared from each mutant and the Parent, and determined their kinetic parameters from the Michaelis-Menten equation (**Fig 8**). The apparent pantothenate *K*_m_ values of the mutant clones are 26–609-fold higher (95% CI exclude 0) than that of the Parent line. The maximal velocity (*V*_max_) of pantothenate phosphorylation by lysates prepared from PanOH-A, PanOH-B and CJ-A are also significantly higher (95% CI exclude 0) than that of the Parent. To eliminate the possibility that the elevated *V*_max_ values are due to increased *Pf*PanK1 levels in the mutant parasites, we used DIA-MS to compare the *Pf*PanK1 level in each of the mutant lines with that of the Parent parasite. We found that *Pf*PanK1 levels are unchanged in the CJ-A line (95% CI include 0) and are only elevated by 26 ± 5% and 27 ± 4% (mean ± SEM) in PanOH-A and PanOH-B, respectively (**S3 Fig**). This modest (or lack of an) increase in *Pf*PanK1 levels is insufficient to explain the larger increases in *V*_max_ values observed in lysates prepared from the mutant lines, as the increase in protein levels would need to be ~20-fold for PanOH-A and ~2-fold for PanOH-B to account for the altered *V*_max_ values. We have therefore not adjusted the observed *V*_max_ values to account for these small increases in the *Pf*PanK1 levels. We also calculated the *Pf*PanK relative specificity constant for each parasite line, which indicates the catalytic efficiency of each variant of *Pf*PanK relative to that of the Parent line. The relative specificity constant obtained for PanOH-A (0.74 ± 0.04, mean ± SEM) is not significantly different (95% CI include 0) from that of the Parent. However, those of PanOH-B (0.058 ± 0.004, mean ± SEM) and CJ-A (0.019 ± 0.005, mean ± SEM) are significantly lower (95% CI exclude 0). These data are consistent with all three *Pf*PanK1 mutations observed reducing the enzyme’s affinity for pantothenate, although the associated increase in the enzyme *V*_max_ compensates for the reduced affinity: fully in the PanOH-A clone, but to a much lesser extent in PanOH-B and CJ-A (resulting in a 17-fold and 52-fold reduction in the enzyme’s catalytic efficiency, respectively).

### CJ-A requires a higher extracellular pantothenate concentration

**Fig 9:**
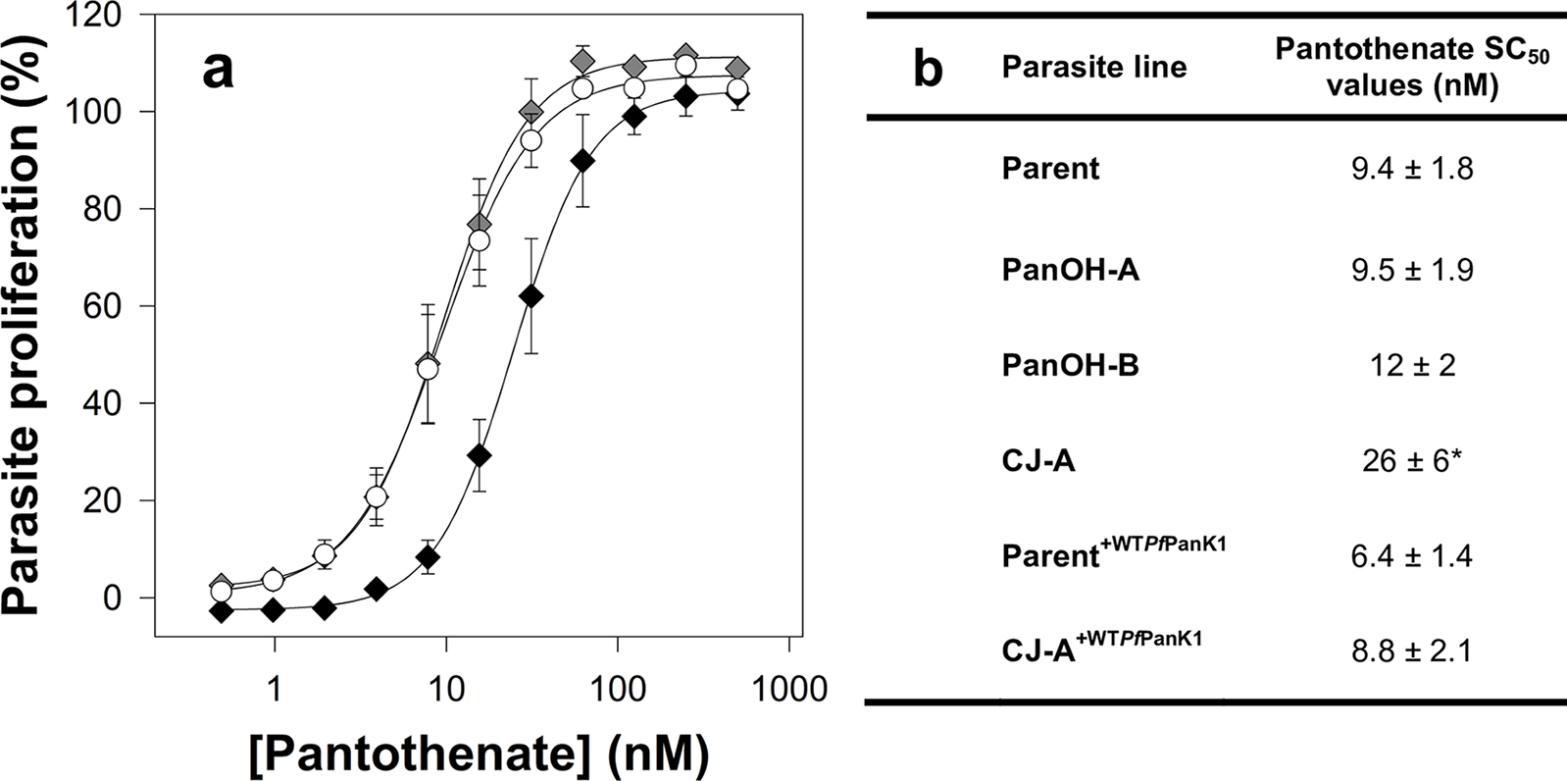
The pantothenate requirement of the different parasite lines observed in this study. (a) Percentage proliferation of parasites grown in different pantothenate concentrations. Values are averaged from ≥ 3 independent experiments, each carried out in triplicate. Error bars represent SEM and are not visible if smaller than the symbols. For clarity, only data from the Parent (white circles), CJ-A (black diamonds), and CJ-A^+WTPfPanK1^ (grey diamonds) lines are shown. (b) The pantothenate stimulatory concentration 50 (SC_50_) values obtained for Parent, PanOH-A, PanOH-B, CJ-A, Parent^+WTPfPanK1^ and CJ-A^+WTPfPanK1^ line parasites, which is the concentration of pantothenate required in the medium to support parasite proliferation by 50% (with 100% set to parasites grown in complete medium containing 1 *μ*M pantothenate). Errors represent SEM (n ≥ 3). An asterisk indicates that the value is significantly different from that obtained for the Parent line (CJ-A SC_50_ value 95% CI compared to Parent: 2.7 to 30.9). No significant difference was observed between the SC_50_ value of the Parent and those of PanOH-A, PanOH-B, Parent^+WTPfPanK1^ and CJ-A^+WTPfPanK1^ (95% CI compared to Parent: PanOH-A = -6.8 to 7.1, PanOH-B = -3.8 to 9.3, Parent^+WTPfPanK1^ = -9.3 to 3.3 & CJ-A^+WTPfPanK1^ = -7.8 to 6.6).

An extracellular supply of pantothenate is essential for the *in vitro* proliferation of the intraerythrocytic stage of *P*. *falciparum* [8]. Given the impact that the *Pf*PanK1 mutations have on PanK activity (**Fig 8**), we set out to determine whether a higher extracellular concentration of pantothenate is required to support the proliferation of the different mutant clones relative to that required by the Parent line. As observed in **Fig 9A**, the proliferation of the Parent line (white circles) increased as the extracellular pantothenate concentration was increased, reaching the 100% control level (parasites maintained in the presence of 1 *μ*M pantothenate, the concentration of pantothenate in the complete RPMI medium used to maintain all of the parasite cultures, which is within the physiologically relevant range in human blood [38,39]) at approximately 100 nM. In order to compare the extracellular pantothenate requirement between the different lines, we determined the SC_50_ (50% stimulatory concentration; i.e. the concentration of pantothenate required to support parasite proliferation to a level equivalent to 50% of the control level) values for the mutants (with and without complementation) and Parent. From **Fig 9B**, it can be seen that the SC_50_ values of PanOH-A and PanOH-B are not different from that of the Parent line (95% CI include 0). Conversely, as illustrated by the rightward shift in its dose-response curve (black diamonds, **Fig 9A**), the pantothenate SC_50_ of CJ-A is approximately 3-fold higher than that of the Parent (**Fig 9B**; 95% CI = 2.7 to 30.9). Furthermore, consistent with the data from the complementation experiments (**Fig 4**), the SC_50_ value of CJ-A^+WT*Pf*PanK1^ (grey diamonds, **Fig 9A)** is comparable to that of the Parent line and also the control line, Parent^+WT*Pf*PanK1^ (**Fig 9B**; 95% CI include 0), indicating that the episomal expression of wild-type *Pf*PanK1 is sufficient to reverse the phenotypic effects of the mutation.

### N5-trz-C1-Pan and *N*-PE-αMe-PanAm have a different mechanism of action to PanOH and CJ-15,801

**Fig 10:**
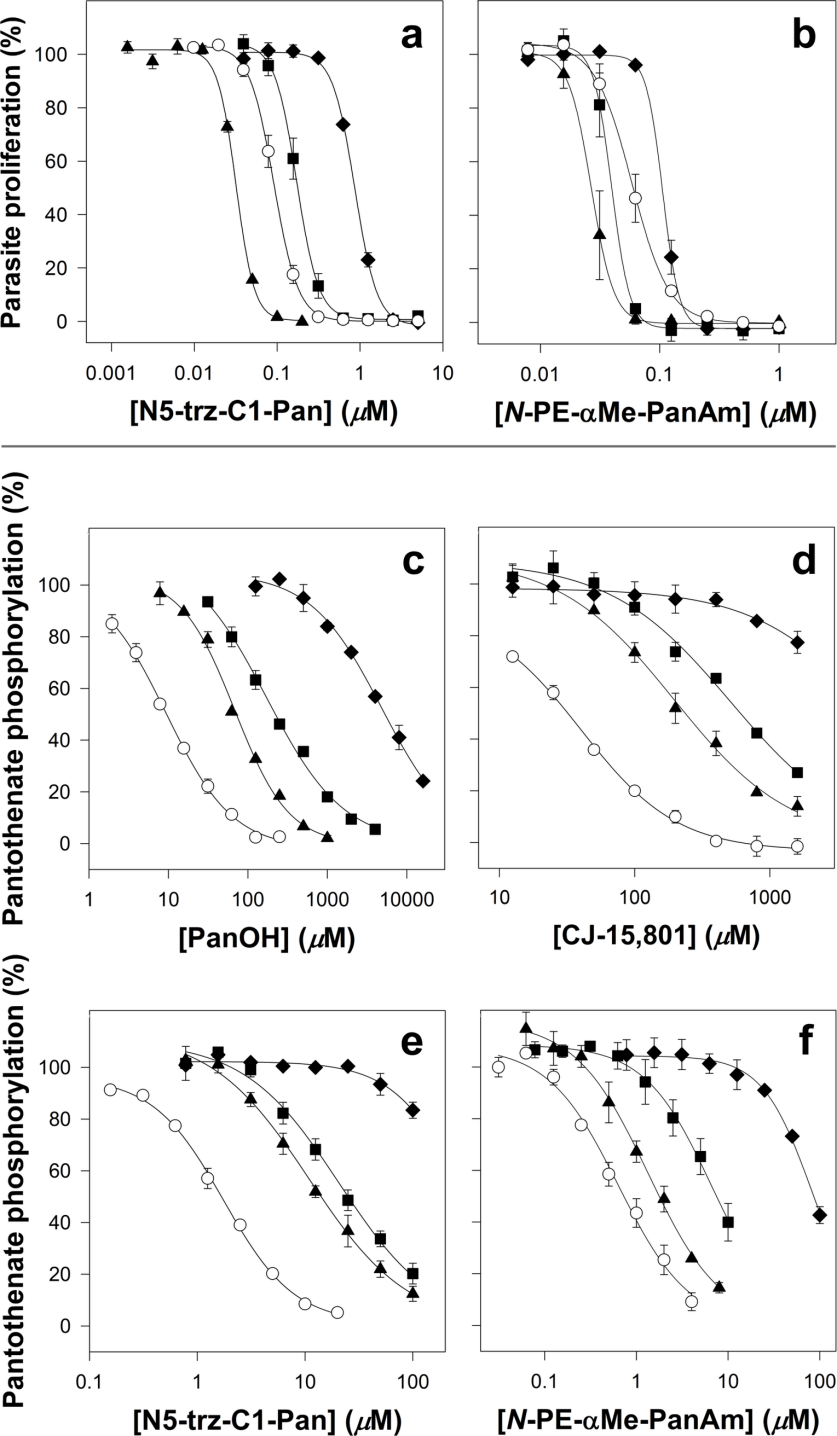
**Percentage parasite proliferation of the different lines in the presence of the pantothenate analogues (a) N5-trz-C1-Pan and (b) *N*-PE-αMe-PanAm, and inhibition of [^14^C]pantothenate phosphorylation in parasite lysates by various pantothenate analogues: (c) PanOH, (d) CJ-15,801, (e) N5-trz-C1-Pan and (f) *N*-PE-αMe-PanAm.** Symbols represent Parent (white circles), PanOH-A (black triangles), PanOH-B (black squares) and CJ-A (black diamonds) parasite lines. The Y-axes of c−f indicate the percentage of total pantothenate phosphorylation. Values are averaged from ≥ 3 independent experiments, each carried out in triplicate for the parasite proliferation assays and duplicate for the phosphorylation assays. Error bars represent SEM and are not visible if smaller than the symbols.

To determine whether the resistance of the clones to PanOH and CJ-15,801 extends to other pantothenate analogues, we tested the mutant clones against the two recently-described, modified pantothenamides with potent antiplasmodial activities, namely N5-trz-C1-Pan [20] and *N*-PE-αMe-PanAm [21]. We found that PanOH-A is 3-fold more *sensitive* to N5-trz-C1-Pan, PanOH-B is 2-fold more resistant and CJ-A is 9-fold more resistant when compared to the Parent line (95% CI exclude 0; **Fig 10A** and **S5 Table**, left side). Similarly, we found that relative to the Parent line, PanOH-A was more sensitive to *N*-PE-αMe-PanAm (~2-fold), while CJ-A is 2-fold more resistant (95% CI exclude 0; **Fig 10B** and **S5 Table**, left side). The sensitivity of PanOH-B to *N*-PE-αMe-PanAm was statistically indistinguishable from that of the Parent line (95% CI = -0.046 to 0.005; **Fig 10B** and **S5 Table**, left side). These results indicate that *Pf*PanK1 can influence the sensitivity of the parasite to multiple antiplasmodial pantothenate analogues. Remarkably, the mutation at position 507 of the *Pf*PanK1 in PanOH-A makes the parasite resistant to the antiplasmodial activity of certain pantothenate analogues (PanOH and CJ-15,801) whilst at the same time *hyper-sensitises* the parasite to pantothenate analogues of a different class (modified pantothenamides, N5-trz-C1-Pan and *N*-PE-αMe-PanAm).

Previous work has shown that, in bacteria, pantothenamides are metabolised by the CoA biosynthetic pathway to form CoA antimetabolites [40], consistent with PanK activity being important for metabolic activation of pantothenamides. Additionally, it has been reported recently that pantothenamides are also phosphorylated by the PanK in *P*. *falciparum* [41], in line with their metabolic activation in bacteria. We therefore set out to determine whether the modified, pantetheinase-resistant, pantothenamides are metabolised and to what extent. In order to do so, we exposed intact Parent line *P*. *falciparum*-infected erythrocytes to N5-trz-C1-Pan (at ~10 × the IC_50_ for 4 h) and subjected lysates from the treated (and control) samples to untargeted LC-MS. Among the metabolites extracted from parasite-infected erythrocytes treated with N5-trz-C1-Pan (but not untreated control samples) were molecules with masses corresponding to phosphorylated N5-trz-C1-Pan ([M-H]^-^ *m/z* 378.1668), a dephospho-CoA analogue of N5-trz-C1-Pan ([M-2H]^2-^ *m/z* 353.6099) and a CoA analogue of N5-trz-C1-Pan ([M-H]^-^ *m/z* 787.1858) as shown in **S4 Fig**. This is consistent with N5-trz-C1-Pan being metabolised within infected erythrocytes to generate a CoA antimetabolite.

Lastly, we investigated the ability of the different pantothenate analogues to inhibit the phosphorylation of [^14^C]pantothenate by parasite lysates prepared from the various mutant clones. These data are shown in **Fig 10C–10F** and **S5 Table**, right side. All of the analogues tested are significantly less effective (95% CI exclude 0) at inhibiting pantothenate phosphorylation by lysates generated from the mutant clones compared to their ability to inhibit pantothenate phosphorylation by lysates prepared from the Parent line. The exception being *N*-PE-αMe-PanAm, which did not reach statistical significance when tested against lysate prepared from PanOH-A. Additionally, the effectiveness of the analogues at inhibiting pantothenate phosphorylation by lysates prepared from the mutant lines all observe the following order: PanOH-A > PanOH-B > CJ-A.

## Discussion

### The putative *Pfpank1* gene codes for a functional pantothenate kinase

When *Pf*PanK1 is compared to type II PanKs from other organisms in a multiple protein sequence alignment, the three nucleotide-binding motifs characteristic of this superfamily can be seen to be conserved in *Pf*PanK1, consistent with it being a functional PanK [7]. However, biochemical confirmation of its putative function as a PanK has not been demonstrated. In this study, we show that mutations in the putative *Pf*PanK1 lead to substantial changes in pantothenate kinase activity (**Fig 8**) providing, for the first time, biochemical evidence that the cytosolic *Pf*PanK1 (**Fig 7**) is a functional pantothenate kinase. Furthermore, the fact that multiple independent experiments aimed at generating parasites resistant to PanOH and CJ-15,801 always selected for mutations in *Pf*PanK1 (**Fig 3A** and **S4 Table**) is consistent with the kinase being the primary PanK involved in the metabolic activation of pantothenate analogues, at least during the intraerythrocytic stage. Although it is intriguing that *Pf*PanK1 is not excluded from the nucleus, this phenomenon has been reported for some PanKs in other organisms [42].

Although it is clear that the *Pf*PanK1 residues at position 95 and 507 are required for normal *Pf*PanK1 activity (**Fig 8**), and the *Pf*PanK1 model structure (**Fig 3B**) shows that both residues are situated adjacent to the enzyme active site, their exact role(s) in modifying the activity of the protein is less obvious. The Gly residue at position 95 is conserved in eukaryotic PanKs [7] and is the residue at the cap of the α2-helix in the inactive conformation of the protein (**S5A Fig**). One possibility is that the change to Ala at this position could affect the structure of the helix and consequently the overall stability of the protein (at least when the protein is in the inactive state), as Gly has been shown to be much better than Ala at conferring structural stability when located at the caps of helices [43]. Alternatively, Gly residues have been shown to be present at a higher frequency in the active sites of some enzymes where they likely confer the flexibility to alternate between open and closed conformations [44]. Although the Gly95 residue is not part of the *Pf*PanK1 active site *per se*, it is within close proximity to the site (**Fig 3B**), and the α2-helix certainly undergoes a conformational change when PanK switches from the inactive conformation to the active one [29]. This conformational change is demonstrated in **S5A Fig**, which shows an overlay of the human PanK3 crystal structures in the active [29] and inactive [45] state (Gly95 in *Pf*PanK1 is equivalent to Gly117 in the human enzyme). It is worth noting that acetyl-CoA (an inhibitor of the enzyme) can only be accommodated in the binding site when the enzyme is in the inactive state, as when the enzyme is in the active state, the α2-helix encroaches on the space occupied by acetyl-CoA (**S5A Fig**). This lends further support to the importance of this helix for the enzyme to transition from the inactive to the active state (and *vice versa*) and, therefore, to the role that Gly95 could play in this process. Either way, these suggestions may explain why a mutation at this position has a greater impact on *Pf*PanK1 function than the mutation at position 507. The Asp residue at position 507 is replaced by a different amino acid (Glu) in most other eukaryotic PanKs, although they are both negatively-charged [7]. A substitution to the uncharged Asn could disrupt any important salt-bridges or hydrogen bonds with the residue at this position. In human PanK3, the amino acid equivalent to Asp507 is Glu354. In the enzyme’s inactive state, Glu354 is within ionic bonding distance of Arg325, which in turn interacts with the 3’-phosphate of acetyl-CoA (**S5B Fig**) [45] and may therefore stabilise the inactive state of the enzyme. Conversely, in the enzyme’s active state, Glu354 and Arg325 are not within ionic bonding distance (**S5B Fig**). If Asp507 in *Pf*PanK1 plays a similar role to that proposed for Glu354 in human PanK3, the change at position 507 to an Asn (abolishing the negative charge) could prevent stabilisation of the inactive state, providing an explanation for the increased activity observed in PanOH-A (**Fig 6**). Determining the crystal structure of *Pf*PanK1 bound with pantothenate may provide a better understanding of the roles these residues play in PanK function.

### *Pf*PanK1 is essential for normal intraerythrocytic proliferation of *P*. *falciparum*

It has been established that almost all of the 4’-phosphopantothenate found in *P*. *falciparum*-infected erythrocytes is generated within the parasite by *Pf*PanK as part of its metabolism into CoA [10], which is in line with *Pf*PanK activity being essential for the parasite’s survival. In the present study, our inability to knock out *Pf*PanK1 (**S2 Fig**) is consistent with the protein being essential for the intraerythrocytic stage of *P*. *falciparum*, although we cannot exclude the unlikely possibility that the regions we targeted for the required double-crossover recombination event are genetically intractable. Our observation that clones PanOH-B and CJ-A, which harbour mutations at position 95 of *Pf*PanK1, can be outcompeted by the Parent parasites in competition assays over approximately 20 intraerythrocytic cycles (**Fig 5**) is consistent with the mutations incurring a fitness cost. This, in turn, indicates that *Pf*PanK1 is essential for normal parasite development, at least during the blood stage of its lifecycle. It was also found here that clone CJ-A requires an approximately 3-fold higher extracellular pantothenate concentration in order to survive (**Fig 9**), coinciding with this clone having the *Pf*PanK with the highest *K*_m_. This is not surprising given the reported importance for the substrate concentration to exceed the enzyme *K*_m_ for optimal enzyme efficiency [46,47]. More importantly, the requirement by this clone for a higher concentration of extracellular pantothenate is also congruent with *Pf*PanK1 being essential for the normal development of *P*. *falciparum* during its asexual blood stage.

Our observation that *Pf*PanK1 is essential for normal parasite growth during the blood stage is at odds with recent reports that show both PanK1 and PanK2 from *Plasmodium yoelli* and *Plasmodium berghei* are dispensable during the blood stage of those parasites [48–50]. However, both of these murine malaria parasite species preferentially infect reticulocytes [51,52]. Unlike the mature erythrocytes preferred by *P*. *falciparum*, reticulocytes have been shown to provide a rich pool of nutrients for the parasite, allowing the murine parasites to survive metabolic or genetic changes that would have been deleterious in *P*. *falciparum* [53]. It is therefore conceivable that unlike the condition faced by *P*. *falciparum*, the reticulocyte-residing parasites are able to salvage sufficient CoA or CoA intermediates from the host cell for their survival, rendering the two PanK proteins dispensable during their intraerythrocytic stage, a possibility acknowledged by the authors of the *P*. *berghei* study [49].

### PanOH and CJ-15,801 share a common mechanism of action

**Fig 11:**
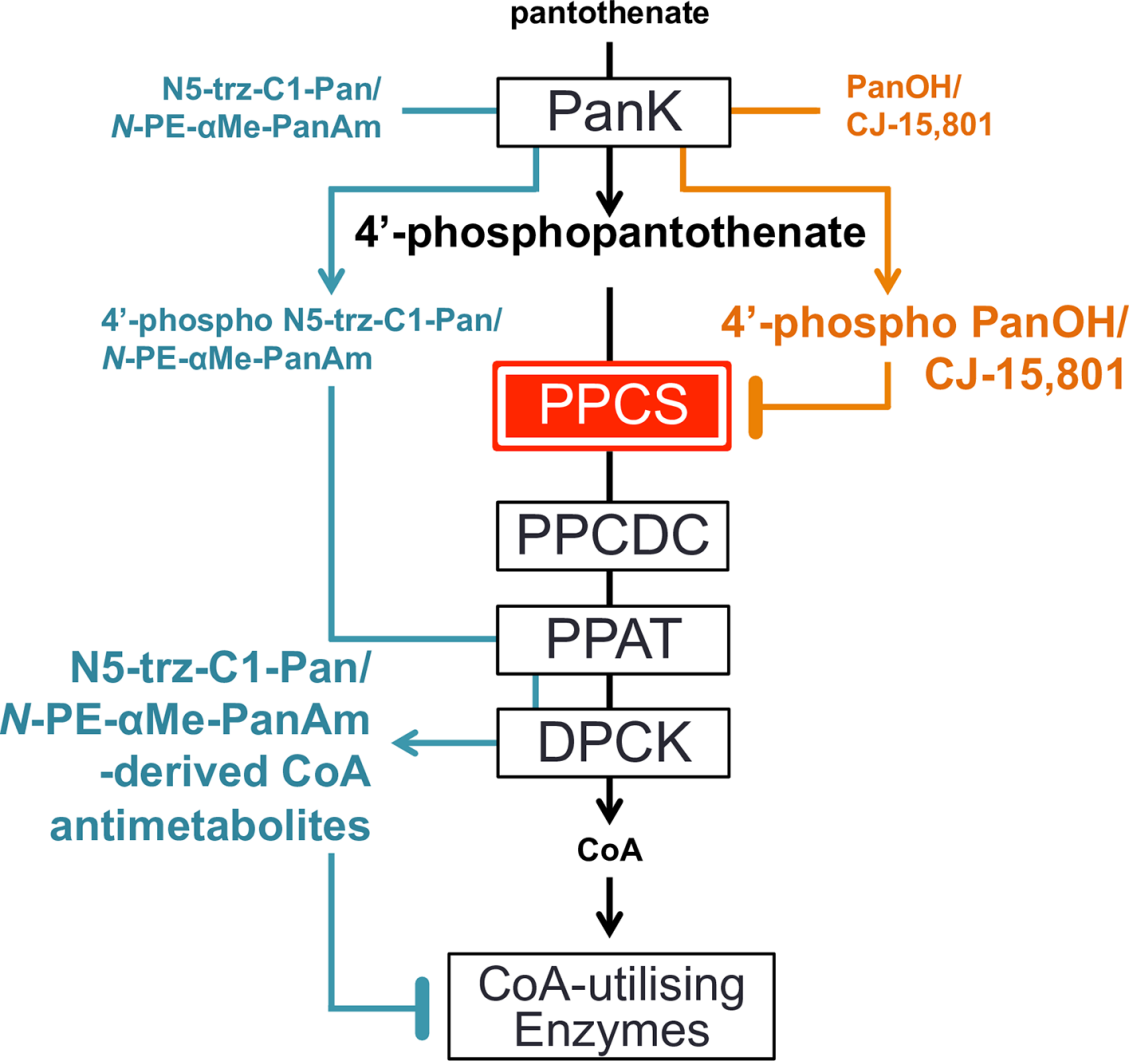
The proposed antiplasmodial mechanisms of action of various pantothenate analogues in *Plasmodium falciparum*. PanOH and CJ-15,801 (orange) are phosphorylated by *Pf*PanK and their phosphorylated forms accumulate (indicated by the bigger font) and then competitively inhibit *Pf*PPCS. The step mediated by *Pf*PPCS has been hypothesised to be a pathway flux control point (illustrated by the red box) in the CoA biosynthesis pathway. On the other hand, we propose that although *N*-PE-αMe-PanAm and N5-trz-C1-Pan (cyan) are also phosphorylated by *Pf*PanK, they then bypass *Pf*PPCS and *Pf*PPCDC (as shown previously in bacteria) and rejoin the pathway as substrates of *Pf*PPAT and then *Pf*DPCK, resulting in the generation and accumulation of CoA antimetabolites. These CoA antimetabolites exert their antiplasmodial activity by inhibiting CoA-dependent processes.

We have presented data consistent with the observed *Pf*PanK1 mutations being the genetic basis for the PanOH and CJ-15,801 resistance phenotypes observed in all of the drug-pressured clones we generated (**Fig 4**). As shown by the data presented in **Fig 10**, both PanOH and CJ-15,801 inhibited pantothenate phosphorylation by the mutated *Pf*PanK1 proteins less effectively than their inhibition of pantothenate phosphorylation by the wild-type *Pf*PanK1 (the order of their IC_50_ values is Parent < PanOH-A < PanOH-B < CJ-A). Importantly, this order is also reflected in the level of resistance of the mutant clones to these two analogues, although the magnitude is not preserved (**Fig 2** and **S3 Table**). These data are consistent with *Pf*PanK1 being involved in the antiplasmodial activity of these analogues either as a target or a metabolic activator. Previous studies have demonstrated that the antiplasmodial activity of both PanOH and CJ-15,801 involves the inhibition of pantothenate phosphorylation by *Pf*PanK [9,15]. More specifically, PanOH has been shown to inhibit *Pf*PanK-mediated pantothenate phosphorylation by serving as its substrate [54]. CJ-15,801 is also likely to be a substrate of *Pf*PanK, especially since it has been shown to be phosphorylated by the *S*. *aureus* PanK [55], another type II PanK [56]. In addition, the same study showed that the second enzyme in the CoA biosynthetic pathway, PPCS, subsequently accepts phosphorylated CJ-15,801 as a substrate, and performs the first step of the PPCS reaction (cytidylylation) on it. However, unlike what happens with 4’-phosphopantothenate, this cytidylylated phospho-CJ-15,801 acts as a tight-binding, dead-end inhibitor of the enzyme [55]. A separate study also concluded that PanOH targets the PPCS enzyme in *Escherichia coli* and *Mycobacterium tuberculosis* [57]. Based on the data generated in this study and the published reports that PanOH and CJ-15,801 both inhibit PPCS in other systems, we propose a similar mechanism of action for these compounds in *P*. *falciparum*, whereby they are phosphorylated by *Pf*PanK1 and subsequently block *Pf*PPCS as dead-end inhibitors (**Fig 11**). The overlapping pattern of cross-resistance between the two compounds (**Fig 2**) is also in line with them having a similar mechanism of action. We propose that the observed resistance to PanOH and CJ-15,801 is due to the mutated *Pf*PanK1 having a reduced capacity to phosphorylate these analogues relative to pantothenate. This would have the effect of reducing the amount of phosphorylated PanOH or CJ-15,801 generated relative to 4’-phosphopantothenate, thereby allowing the parasites to survive at higher concentrations of the drugs. Furthermore, our observation that the mutant clones have comparable levels of PanOH and CJ-15,801 resistance (**Fig 2** and **S3 Table**), despite having *Pf*PanK1 proteins of vastly different efficiency (**Fig 8**), is likely due to the pathway flux control at the *Pf*PPCS-mediated step in the CoA biosynthetic pathway of *P*. *falciparum*, as shown in a previous study [32].

### N5-trz-C1-Pan is converted into an antiplasmodial CoA analogue – *N*-PE-αMe-PanAm likely shares the same mechanism of action

N5-trz-C1-Pan and *N*-PE-αMe-PanAm are pantothenamide-mimics that harbour modifications designed to prevent them from being substrates of pantetheinase, thereby preventing their degradation: *N*-PE-αMe-PanAm is methylated at the α-carbon [21] while N5-trz-C1-Pan harbours a triazole instead of the labile amide [20]. Our LC-MS data clearly show that N5-trz-C1-Pan is converted into a CoA antimetabolite (**S4 Fig**), and it is therefore likely to go on to inhibit/inactivate CoA-utilising enzymes, killing the parasite. Such a mechanism has previously been put forward to explain the antibiotic activity of two prototypical pantothenamides (N5-Pan and N7-Pan) whereby the compounds are phosphorylated by PanK and subsequently metabolised by PPAT and DPCK to generate analogues of CoA (ethyldethia-CoA and butyldethia-CoA) [40,58,59]. These CoA analogues then mediate their antibacterial effect/s primarily by inhibiting/inactivating CoA-requiring enzymes and acyl carrier proteins [40,58,59]. Similarly, phospho-N5-trz-C1-Pan is not expected to interact with *Pf*PPCS because it lacks the carboxyl group (**Fig 1**) required for the nucleotide activation by nucleotide transfer [55]. It is therefore expected to bypass the *Pf*PPCS and *Pf*PPCDC steps of the CoA biosynthetic pathway on its way to being converted into the CoA antimetabolite version of N5-trz-C1-Pan (**Fig 11**).

Furthermore, the antiplasmodial activity rank order of N5-trz-C1-Pan against the various mutant clones is very similar to that of *N*-PE-αMe-PanAm – with PanOH-A being hypersensitive to both compounds, CJ-A resistant to both and PanOH-B, by comparison, exhibiting only small changes in sensitivity to the two compounds (**Fig 10A and 10B**) – and is starkly different to those of PanOH and CJ-15,801 (**Fig 2**). These are congruent with (i) the antiplasmodial mechanism of action of *N*-PE-αMe-PanAm being similar to that of N5-trz-C1-Pan and (ii) the antiplasmodial mechanism of action of *N*-PE-αMe-PanAm and N5-trz-C1-Pan being different to that of PanOH and CJ-15,801.

The order of antiplasmodial activity of N5-trz-C1-Pan and *N*-PE-αMe-PanAm against the mutant clones can be explained on the basis of (i) the difference in the rate of *Pf*PanK1 activity in the various clones at the concentrations of pantothenate and N5-trz-C1-Pan / *N*-PE-αMe-PanAm used (**Fig 6**), (ii) the fact that the *Pf*PPCS-mediated step imposes pathway flux control [32] and (iii) the fact that this pathway flux control is bypassed by N5-trz-C1-Pan (and almost certainly also by *N*-PE-αMe-PanAm) *en route* to its conversion into a CoA antimetabolite. As seen in **Fig 6**, in the presence of 2 *μ*M pantothenate (a similar concentration to the 1 *μ*M present in the antiplasmodial assay), the pantothenate phosphorylation rate of the different clones has the following rank order: PanOH-A > Parent > PanOH-B > CJ-A, approximately the inverse of the antiplasmodial IC_50_ values of N5-trz-C1-Pan and *N*-PE-αMe-PanAm (described above). Therefore, PanOH-A, for example, would be expected to generate more 4’-phosphopantothenate and phosphorylated N5-trz-C1-Pan (or *N*-PE-αMe-PanAm), based on the assumption that the mutation also leads to increased phosphorylation activity towards the pantothenate analogues (point i). Whilst the increased levels of 4’-phosphopantothenate would not be expected to result in a concomitant increase in CoA (due to the pathway flux control at *Pf*PPCS; point ii), the increased production of phospho-N5-trz-C1-Pan would result in increased levels of the N5-trz-C1-Pan CoA antimetabolite as the flux control step is bypassed (point iii). This would explain the increased sensitivity of PanOH-A to both N5-trz-C1-Pan and *N*-PE-αMe-PanAm and also the sensitivity rank order of the other parasite lines.

In conclusion, our study confirms for the first time that *Pf*PanK1 functions as the active pantothenate kinase in the asexual blood stage of *P*. *falciparum*. Our data show that the sites of mutation in *Pf*PanK1 reported here are important residues for normal *Pf*PanK function and are essential for normal intraerythrocytic parasite growth, although further structural and functional studies are required to elucidate their exact role(s). Furthermore, we propose that following phosphorylation by *Pf*PanK1, PanOH and CJ-15,801 compete with 4’-phosphopantothenate and serve as dead-end inhibitors of *Pf*PPCS (depriving the parasite of CoA). In contrast, *N*-PE-αMe-PanAm and N5-trz-C1-Pan are further metabolised (by *Pf*PPAT and *Pf*DPCK) into CoA analogues that kill the parasite by inhibiting CoA-utilising metabolic processes. Finally, we provide the first genetic evidence consistent with pantothenate analogue activation being a critical step in their antiplasmodial activity.

## Supporting information

S1 Materials and methods Additional information about materials and methods used in the study. (DOCX)

S1 Table List of oligonucleotides used in this study. MCS: Multiple Cloning Site.
(DOCX)

S2 Table Sequences of peptides used to identify and quantify the five proteins of interest shown in S3 Fig. ^a^Certainty of peptide identification.
(DOCX)

S3 Table Inhibition of the proliferation of Parent, PanOH-A, PanOH-B, CJ-A, Parent^+WT*Pf*PanK1^, PanOH-A^+WT*Pf*PanK1^, PanOH-B^+WT*Pf*PanK1^ and CJ-A^+WT*Pf*PanK1^ parasites by PanOH, CJ-15,801 and chloroquine, as represented by IC_50_ values. Errors represent SEM (n ≥ 3). The averaged CJ-15,801 IC_50_ value for PanOH-B (n = 3) includes a single extrapolated value, because in one experiment the highest concentration tested (800 *μ*M) did not inhibit parasite growth by ≥ 50%. An asterisk indicates that the IC_50_ value of the mutant line is significantly different from that obtained for the Parent line (PanOH IC_50_ 95% CI compared to Parent IC_50_: PanOH-A = 2664 to 3432, PanOH-B = 3553 to 4552 & CJ-A = 6046 to 7456; CJ-15,801 IC_50_ 95% CI compared to Parent IC_50_: PanOH-A = 296 to 436, PanOH-B = 495 to 717 & CJ-A = 688 to 823). The chloroquine IC_50_ values of the different lines are indistinguishable (95% CI compared to Parent IC_50_: PanOH-A = -2.023 to 1.646, PanOH-B = -2.114 to 0.492 & CJ-A = -2.507 to 0.158).
(DOCX)

S4 Table List of non-synonymous single nucleotide polymorphisms found in the PanOH-A, PanOH-B and CJ-A parasite lines as determined by whole genome sequencing and variant calling using the PlaTyPus integrated pipeline. Uppercase letters in the “Codon Change” column denotes the base within the codon that has been altered in the coding sequence before/after the mutation. The deletion in PanOH-B at position 95 is not included here because PlaTyPus is unable to detect insertions-deletions polymorphisms.
(DOCX)

S5 Table Inhibition of the proliferation of Parent, PanOH-A, PanOH-B and CJ-A parasites by N5-trz-C1-Pan and *N*-PE-αMe-PanAm, and the inhibition of [^14^C]pantothenate phosphorylation in parasite lysates (generated from the same lines) by the pantothenate analogues PanOH, CJ-15,801, N5-trz-C1-Pan and *N*-PE-αMe-PanAm, as represented by IC_50_ values. Errors represent SEM (n ≥ 3). An asterisk indicates that the value is significantly different from that obtained for the Parent line (95% CI of N5-trz-C1-Pan proliferation inhibition IC_50_ compared to Parent: PanOH-A = -0.088 to -0.038, PanOH-B = 0.047 to 0.121 & CJ-A = 0.715 to 0.824; 95% CI of PE-αMe-PanAm proliferation inhibition IC_50_ compared to Parent: PanOH-A = -0.060 to -0.008 & CJ-A = 0.024 to 0.076; 95% CI of PanOH phosphorylation inhibition IC_50_ compared to Parent: PanOH-A = 46 to 69, PanOH-B = 71 to 263 & CJ-A = 4531 to 5300; 95% CI of CJ-15,801 phosphorylation inhibition IC_50_ compared to Parent: PanOH-A = 10 to 309 & PanOH-B = 330 to 621; 95% CI of N5-trz-C1-Pan phosphorylation inhibition IC_50_ compared to Parent: PanOH-A = 6.5 to 13 & PanOH-B = 9.8 to 30; 95% CI of *N*-PE-αMe-PanAm phosphorylation inhibition IC_50_ compared to Parent: PanOH-B = 3.6 to 9.0 & CJ-A = 75 to 97).
(DOCX)

S1 Fig Quantitative PCR analysis of gDNA samples extracted from the three competition assay cultures generated in the study, Parent vs PanOH-A, Parent vs PanOH-B and Parent vs CJ-A (Fig 5). Data are shown as the proportions of mutant gDNA (out of a 100% total) at week 0 and week 6. Values are averaged from two independent competition assays, each analysed by qPCR in duplicate. Error bars represent range/2.
(TIF)

S2 Fig The attempt at knocking-out *Pfpank1* in wild-type 3D7 parasites through homologous integration. (a) Schematic representations of the Δ*Pfpank1*-pCC-1 construct and the wild-type *Pfpank1* gene locus before and after either a homologous double crossover integration of the *hdhfr* cassette of Δ*Pfpank1*-pCC-1 or a 5’/3’ single crossover homologous integration of the Δ*Pfpank1*-pCC-1 construct. The positions of *Afl*II restriction sites are indicated. The 5’ *Pfpank1* and 3’ *Pfpank1* homologous flanks are indicated by translucent blue boxes. (b) Southern blot of *Afl*II-digested gDNA extracted from wild-type parasites and from Δ*Pfpank1*-pCC-1-transfectants resistant to WR99210 or both WR99210 and 5-fluorocytosine (5-FC) after one or two rounds of WR99210 cycling. (i) and (ii) represent independently-selected drug-resistant cultures. The blot was probed with the 3’ *Pfpank1* flank (as indicated by the black bar in (a)). The probe hybridised to fragments that correspond to the 3D7 wild-type (3.3 kb) and the plasmid (8.9 kb). The fragment that is consistent with a homologous double crossover-disrupted locus (3.8 kb) was not detected in either independent culture. *hdhfr*: human dihydrofolate reductase—conveys resistance to WR99210. *Scfcu*: *Saccharomyces cerevisiae* cytosine deaminase/phosphoribosyl transferase—conveys sensitivity to 5-FC. (c) PCR confirmation that a *Pfpank1* knockout event cannot be detected, even in a small sub-population of parasites. Samples from the same parasite populations shown in (b) were used to generate DNA templates that were then used with primers selected to amplify a product of 1.9 kb from the wild-type sequence and a 3.6 kb product from parasites with the *Pfpank1* gene knocked out (and replaced with the *hdhfr* gene). A PCR product consistent with the size expected for amplification from the wild-type sequence was observed (black arrow). The identity of this product was confirmed by sequencing. All the reactions generated an approximately 1.1 kb PCR product and some reactions, under certain conditions, produced fainter PCR products likely due to non-specific primer binding. Importantly, no product of a size consistent with that expected for a *Pfpank1* knockout (3.6 kb) was observed in any of the samples. These data, therefore, are consistent with those presented in the Southern blot (b).
(TIF)

S3 Fig **Abundance of (a) *Pf*PanK1 and (b) various control proteins (*Pf*PanK2, *Pf*HSP70, *Pf*HK and *Pf*α-tubulin) in Parent, PanOH-A, PanOH-B and CJ-A line trophozoites.** Protein levels are determined by LC-MS/MS followed by DIA analysis. Values are averaged from ≥ 3 independent parasite preparations and error bars represent SEM. An asterisk indicates that the protein peak intensity measured for a mutant line is significantly different from that obtained for the Parent line (95% CI of *Pf*PanK1 protein level compared to Parent: PanOH-A = 3.87 × 10^3^ to 1.18 × 10^5^ & PanOH-B = 8.86 × 10^3^ to 1.18 × 10^5^). The protein abundance of *Pf*PanK1 in CJ-A (95% CI compared to Parent = -3.38 × 10^4^ to 1.01 × 10^5^) and those of the housekeeping proteins in all three mutant lines are indistinguishable from the Parent line levels (95% CI for *Pf*PanK2 level compared to Parent: PanOH-A = -1.81 × 10^4^ to 2.53 × 10^4^, PanOH-B = -1.52 × 10^4^ to 2.25 × 10^4^ & CJ-A = -3.43 × 10^4^ to 1.99 × 10^4^; 95% CI for *Pf*HSP70 level compared to Parent: PanOH-A = -8.98 × 10^7^ to 9.72 × 10^7^, PanOH-B = -6.92 × 10^7^ to 6.27 × 10^7^ & CJ-A = -1.45 × 10^8^ to 1.45 × 10^8^; 95% CI for *Pf*HK level compared to Parent: PanOH-A = -4.41 × 10^5^ to 2.79 × 10^6^, PanOH-B = -1.18 × 10^5^ to 2.47 × 10^6^ & CJ-A = -2.42 × 10^6^ to 2.13 × 10^6^; 95% CI for *Pf*α-tubulin level compared to Parent: PanOH-A = -5.57 × 10^6^ to 7.45 × 10^6^, PanOH-B = -4.47 × 10^6^ to 4.25 × 10^6^ & CJ-A = -1.18 × 10^7^ to 2.22 × 10^6^).
(TIF)

S4 Fig **Extracted ion chromatograms (i) and mass spectra (ii) of N5-trz-C1-Pan (a) and downstream metabolites (b–d).** Extracted ion chromatograms (i) show the relative intensity of each LC peak. Red lines represent metabolites extracted from N5-trz-C1-Pan-treated parasites and blue lines represent those extracted from DMSO-treated parasite control samples, each carried out in quadruplicate. High resolution mass spectra (ii) of each compound ionised in negative mode: (a) N5-trz-C1-Pan (C_14_H_26_N_4_O_3_). Theoretical *m/z* = 297.1932. Observed *m/z* = 297.1942. Δppm = 3.36. (b) Phosphorylated N5-trz-C1-Pan (C_14_H_27_N_4_O_6_P). Theoretical *m/z* = 377.1595. Observed *m/z* = 377.1594. Δppm = -0.27. (c) Dephospho-CoA N5-trz-C1-Pan analogue (C_24_H_39_N_9_O_12_P_2_). Theoretical *m/z* = 352.6024. Observed *m/z* = 352.6024. Δppm = 0.0. (d) CoA N5-trz-C1-Pan analogue (C_24_H_40_N_9_O_15_P_3_). Theoretical *m/z* = 786.1784. Observed *m/z* = 786.1780. Δppm = -0.51. Data shown are from a single experiment representative of two independent experiments.
(TIF)

S5 Fig The human PanK3 protein structure in its inactive conformation (yellow; PDB ID: 3MK6) overlaid onto its active conformation (blue; PDB ID: 5KPR). The amino acid residues of the human protein at positions 117 (indicated by the spheres in a) and 354 (side chains shown in b) correspond to the mutated residues of *Pf*PanK1 reported in this study (at positions 95 and 507, respectively). (a) The red arrow indicates the change in the conformation of the α2-helix between the inactive and active states. The altered configuration in the active state transitions the mutated glycine that corresponds to position 117 away from the end cap of the α2-helix. (b) Dashed grey lines (inactive conformation) and the solid arrow (active conformation) represent distances between residue side chains and/or acetyl-CoA (in Å) in PanK3. A relay of interactions between Glu354, Arg325 and the 3’-phosphate of acetyl-CoA may stabilise the inactive state of the enzyme. However, in the protein’s active conformation, Glu354 and Arg325 are not within bonding distance (≥ 4.4 Å).
(TIF)

## References

[1] WHO. World Malaria Report 2016. World Health Organization 2016.

[2] Dondorp AM, Nosten F, Yi P, Das D, Phyo AP, Tarning J, et al Artemisinin resistance in *Plasmodium falciparum* malaria. N Engl J Med. 2009;361: 455–467. doi: 10.1056/NEJMoa0808859 19641202 10.1056/NEJMoa0808859 PMC3495232

[3] Sutherland CJ, Lansdell P, Sanders M, Muwanguzi J, van Schalkwyk DA, Kaur H, et al *pfk13*-independent treatment failure in four imported cases of *Plasmodium falciparum* malaria given artemether-lumefantrine in the United Kingdom. Antimicrob Agents Chemother. 2017;61: e02382–16. doi: 10.1128/AAC.02382-16 28137810 10.1128/AAC.02382-16 PMC5328508

[4] Lu F, Culleton R, Zhang M, Ramaprasad A, Seidlein von L, Zhou H, et al Emergence of indigenous artemisinin-resistant *Plasmodium falciparum* in Africa. N Engl J Med. 2017;376: 991–993. doi: 10.1056/NEJMc1612765 28225668 10.1056/NEJMc1612765

[5] Wells TNC, Hooft van Huijsduijnen R, Van Voorhis WC. Malaria medicines: a glass half full? Nat Rev Drug Discov. 2015;14: 424–442. doi: 10.1038/nrd4573 26000721 10.1038/nrd4573

[6] Spry C, Kirk K, Saliba KJ. Coenzyme A biosynthesis: an antimicrobial drug target. Fems Microbiol Rev. 2008;32: 56–106. doi: 10.1111/j.1574-6976.2007.00093.x 18173393 10.1111/j.1574-6976.2007.00093.x

[7] Spry C, van Schalkwyk DA, Strauss E, Saliba KJ. Pantothenate utilization by *Plasmodium* as a target for antimalarial chemotherapy. Infect Disord Drug Targets. 2010;10: 200–216. 20334619 10.2174/187152610791163390

[8] Divo AA, Geary TG, Davis NL, Jensen JB. Nutritional requirements of *Plasmodium falciparum* in culture. I. Exogenously supplied dialyzable components necessary for continuous growth. J Protozool. 1985;32: 59–64. 3886898 10.1111/j.1550-7408.1985.tb03013.x

[9] Saliba KJ, Ferru I, Kirk K. Provitamin B5 (pantothenol) inhibits growth of the intraerythrocytic malaria parasite. Antimicrob Agents Chemother. 2005;49: 632–637. doi: 10.1128/AAC.49.2.632-637.2005 15673744 10.1128/AAC.49.2.632-637.2005 PMC547364

[10] Saliba KJ, Horner HA, Kirk K. Transport and metabolism of the essential vitamin pantothenic acid in human erythrocytes infected with the malaria parasite *Plasmodium falciparum*. J Biol Chem. 1998;273: 10190–10195. 9553068 10.1074/jbc.273.17.10190

[11] Saliba KJ, Kirk K. H^+^-coupled pantothenate transport in the intracellular malaria parasite. J Biol Chem. 2001;276: 18115–18121. doi: 10.1074/jbc.M010942200 11278793 10.1074/jbc.M010942200

[12] Genschel U. Coenzyme A biosynthesis: reconstruction of the pathway in archaea and an evolutionary scenario based on comparative genomics. Mol Biol Evol. 2004;21: 1242–1251. doi: 10.1093/molbev/msh119 15014152 10.1093/molbev/msh119

[13] Pinney JW, Shirley MW, McConkey GA, Westhead DR. metaSHARK: software for automated metabolic network prediction from DNA sequence and its application to the genomes of *Plasmodium falciparum* and *Eimeria tenella*. Nucleic Acids Res. 2005;33: 1399–1409. doi: 10.1093/nar/gki285 15745999 10.1093/nar/gki285 PMC552966

[14] Bozdech Z, Llinas M, Pulliam BL, Wong ED, Zhu JC, DeRisi JL. The transcriptome of the intraerythrocytic developmental cycle of *Plasmodium falciparum*. PLoS Biol. 2003;1: 85–100. doi: 10.1371/journal.pbio.0000005 12929205 10.1371/journal.pbio.0000005 PMC176545

[15] Saliba KJ, Kirk K. CJ-15,801, a fungal natural product, inhibits the intraerythrocytic stage of *Plasmodium falciparum* in vitro via an effect on pantothenic acid utilisation. Mol Biochem Parasitol. 2005;141: 129–131. doi: 10.1016/j.molbiopara.2005.02.003 15811536 10.1016/j.molbiopara.2005.02.003

[16] Spry C, Chai CLL, Kirk K, Saliba KJ. A class of pantothenic acid analogs inhibits *Plasmodium falciparum* pantothenate kinase and represses the proliferation of malaria parasites. Antimicrob Agents Chemother. 2005;49: 4649–4657. doi: 10.1128/AAC.49.11.4649-4657.2005 16251308 10.1128/AAC.49.11.4649-4657.2005 PMC1280137

[17] Spry C, Macuamule C, Lin Z, Virga KG, Lee RE, Strauss E, et al Pantothenamides are potent, on-target inhibitors of *Plasmodium falciparum* growth when serum pantetheinase is inactivated. PLoS One. 2013;8: e54974 doi: 10.1371/journal.pone.0054974 23405100 10.1371/journal.pone.0054974 PMC3566143

[18] de Villiers M, Macuamule C, Spry C, Hyun Y-M, Strauss E, Saliba KJ. Structural modification of pantothenamides counteracts degradation by pantetheinase and improves antiplasmodial activity. ACS Med Chem Lett. 2013;4: 784–789. doi: 10.1021/ml400180d 24900746 10.1021/ml400180d PMC4027574

[19] Pett HE, Jansen PAM, Hermkens PHH, Botman PNM, Beuckens-Schortinghuis CA, Blaauw RH, et al Novel pantothenate derivatives for anti-malarial chemotherapy. Malar J. 2015;14: 169 doi: 10.1186/s12936-015-0673-8 25927675 10.1186/s12936-015-0673-8 PMC4425855

[20] Howieson VM, Tran E, Hoegl A, Fam HL, Fu J, Sivonen K, et al Triazole substitution of a labile amide bond stabilizes pantothenamides and improves their antiplasmodial potency. Antimicrob Agents Chemother. 2016;60: 7146–7152. doi: 10.1128/AAC.01436-16 27645235 10.1128/AAC.01436-16 PMC5118993

[21] Macuamule CJ, Tjhin ET, Jana CE, Barnard L, Koekemoer L, de Villiers M, et al A pantetheinase-resistant pantothenamide with potent, on-target, and selective antiplasmodial activity. Antimicrob Agents Chemother. 2015;59: 3666–3668. doi: 10.1128/AAC.04970-14 25845876 10.1128/AAC.04970-14 PMC4432145

[22] Allen RJ, Kirk K. *Plasmodium falciparum* culture: The benefits of shaking. Mol Biochem Parasitol. 2010;169: 63–65. doi: 10.1016/j.molbiopara.2009.09.005 19766147 10.1016/j.molbiopara.2009.09.005

[23] Rosario V. Cloning of naturally occurring mixed infections of malaria parasites. Science. 1981;212: 1037–1038. 7015505 10.1126/science.7015505

[24] Tjhin ET, Staines HM, van Schalkwyk DA, Krishna S, Saliba KJ. Studies with the *Plasmodium falciparum* hexokinase reveal that PfHT limits the rate of glucose entry into glycolysis. FEBS Lett. 2013;587: 3182–3187. doi: 10.1016/j.febslet.2013.07.052 23954294 10.1016/j.febslet.2013.07.052

[25] Sewell AL, Villa MVJ, Matheson M, Whittingham WG, Marquez R. Fast and flexible synthesis of pantothenic acid and CJ-15,801. Org Lett. 2011;13: 800–803. doi: 10.1021/ol103114w 21250753 10.1021/ol103114w

[26] Awuah E, Ma E, Hoegl A, Vong K, Habib E, Auclair K. Exploring structural motifs necessary for substrate binding in the active site of *Escherichia coli* pantothenate kinase. Bioorg Med Chem. 2014;22: 3083–3090. doi: 10.1016/j.bmc.2014.04.030 24814884 10.1016/j.bmc.2014.04.030 PMC5233448

[27] Manary MJ, Singhakul SS, Flannery EL, Bopp SE, Corey VC, Bright AT, et al Identification of pathogen genomic variants through an integrated pipeline. BMC Bioinformatics. 2014;15: 63 doi: 10.1186/1471-2105-15-63 24589256 10.1186/1471-2105-15-63 PMC3945619

[28] Hayward R, Saliba KJ, Kirk K. *pfmdr1* mutations associated with chloroquine resistance incur a fitness cost in *Plasmodium falciparum*. Mol Microbiol. 2005;55: 1285–1295. doi: 10.1111/j.1365-2958.2004.04470.x 15686571 10.1111/j.1365-2958.2004.04470.x

[29] Subramanian C, Yun MK, Yao J, Sharma LK, Lee RE, White SW, et al Allosteric regulation of mammalian pantothenate kinase. J Biol Chem. 2016;291: 22302–22314. doi: 10.1074/jbc.M116.748061 27555321 10.1074/jbc.M116.748061 PMC5064008

[30] Kelley LA, Mezulis S, Yates CM, Wass MN, Sternberg MJE. The Phyre2 web portal for protein modeling, prediction and analysis. Nat Protoc. 2015;10: 845–858. doi: 10.1038/nprot.2015.053 25950237 10.1038/nprot.2015.053 PMC5298202

[31] Spry C, Saliba KJ, Strauss E. A miniaturized assay for measuring small molecule phosphorylation in the presence of complex matrices. Anal Biochem. 2014;451: 76–78. doi: 10.1016/j.ab.2013.12.010 24333332 10.1016/j.ab.2013.12.010

[32] Spry C, Saliba KJ. The human malaria parasite *Plasmodium falciparum* is not dependent on host coenzyme A biosynthesis. J Biol Chem. 2009;284: 24904–24913. doi: 10.1074/jbc.M109.025312 19584050 10.1074/jbc.M109.025312 PMC2757193

[33] Creek DJ, Chua HH, Cobbold SA, Nijagal B, MacRae JI, Dickerman BK, et al Metabolomics-based screening of the Malaria Box reveals both novel and established mechanisms of action. Antimicrob Agents Chemother. 2016;60: 6650–6663. doi: 10.1128/AAC.01226-16 27572396 10.1128/AAC.01226-16 PMC5075070

[34] Creek DJ, Jankevics A, Burgess KEV, Breitling R, Barrett MP. IDEOM: an Excel interface for analysis of LC-MS-based metabolomics data. Bioinformatics. 2012;28: 1048–1049. doi: 10.1093/bioinformatics/bts069 22308147 10.1093/bioinformatics/bts069

[35] Siddiqui G, Srivastava A, Russell AS, Creek DJ. Multi-omics based identification of specific biochemical changes associated with PfKelch13-mutant artemisinin-resistant *Plasmodium falciparum*. J Infect Dis. 2017;215: 1435–1444. doi: 10.1093/infdis/jix156 28368494 10.1093/infdis/jix156

[36] Escher C, Reiter L, MacLean B, Ossola R, Herzog F, Chilton J, et al Using iRT, a normalized retention time for more targeted measurement of peptides. Proteomics. 2012;12: 1111–1121. doi: 10.1002/pmic.201100463 22577012 10.1002/pmic.201100463 PMC3918884

[37] Foley M, Tilley L. Quinoline antimalarials: mechanisms of action and resistance and prospects for new agents. Pharmacol Ther. 1998;79: 55–87. 9719345 10.1016/s0163-7258(98)00012-6

[38] Eissenstat BR, Wyse BW, Hansen RG. Pantothenic acid status of adolescents. Am J Clin Nutr. 1986;44: 931–937. 3788840 10.1093/ajcn/44.6.931

[39] Wittwer CT, Schweitzer C, Pearson J, Song WO, Windham CT, Wyse BW, et al Enzymes for liberation of pantothenic acid in blood: use of plasma pantetheinase. Am J Clin Nutr. 1989;50: 1072–1078. 2816792 10.1093/ajcn/50.5.1072

[40] Strauss E, Begley TP. The antibiotic activity of *N*-pentylpantothenamide results from its conversion to ethyldethia-coenzyme A, a coenzyme A antimetabolite. J Biol Chem. 2002;277: 48205–48209. doi: 10.1074/jbc.M204560200 12372838 10.1074/jbc.M204560200

[41] de Villiers M, Spry C, Macuamule CJ, Barnard L, Wells G, Saliba KJ, et al Antiplasmodial mode of action of pantothenamides: pantothenate kinase serves as a metabolic activator not as a target. ACS Infect Dis. 2017;3: 527–541. doi: 10.1021/acsinfecdis.7b00024 28437604 10.1021/acsinfecdis.7b00024

[42] Alfonso-Pecchio A, Garcia M, Leonardi R, Jackowski S. Compartmentalization of mammalian pantothenate kinases. PLoS One. 2012;7: e49509 doi: 10.1371/journal.pone.0049509 23152917 10.1371/journal.pone.0049509 PMC3496714

[43] Serrano L, Neira JL, Sancho J, Fersht AR. Effect of alanine versus glycine in *α*-helices on protein stability. Nature. 1992;356: 453–455. doi: 10.1038/356453a0 1557131 10.1038/356453a0

[44] Yan BX, Sun YQ. Glycine residues provide flexibility for enzyme active sites. J Biol Chem. 1997;272: 3190–3194. 9013553 10.1074/jbc.272.6.3190

[45] Leonardi R, Zhang Y-M, Yun MK, Zhou R, Zeng F-Y, Lin W, et al Modulation of pantothenate kinase 3 activity by small molecules that interact with the substrate/allosteric regulatory domain. Chem Biol. 2010;17: 892–902. doi: 10.1016/j.chembiol.2010.06.006 20797618 10.1016/j.chembiol.2010.06.006 PMC2929395

[46] Bennett BD, Kimball EH, Gao M, Osterhout R, Van Dien SJ, Rabinowitz JD. Absolute metabolite concentrations and implied enzyme active site occupancy in *Escherichia coli*. Nat Chem Biol. 2009;5: 593–599. doi: 10.1038/nchembio.186 19561621 10.1038/nchembio.186 PMC2754216

[47] Park JO, Rubin SA, Xu Y-F, Amador-Noguez D, Fan J, Shlomi T, et al Metabolite concentrations, fluxes and free energies imply efficient enzyme usage. Nat Chem Biol. 2016;12: 482–489. doi: 10.1038/nchembio.2077 27159581 10.1038/nchembio.2077 PMC4912430

[48] Hart RJ, Cornillot E, Abraham A, Molina E, Nation CS, Ben Mamoun C, et al Genetic characterization of *Plasmodium* putative pantothenate kinase genes reveals their essential role in malaria parasite transmission to the mosquito. Sci Rep. 2016;6: 33518 doi: 10.1038/srep33518 27644319 10.1038/srep33518 PMC5028760

[49] Srivastava A, Philip N, Hughes KR, Georgiou K, MacRae JI, Barrett MP, et al Stage-specific changes in *Plasmodium* metabolism required for differentiation and adaptation to different host and vector environments. PLoS Pathog. 2016;12: e1006094 doi: 10.1371/journal.ppat.1006094 28027318 10.1371/journal.ppat.1006094 PMC5189940

[50] Bushell E, Gomes AR, Sanderson T, Anar B, Girling G, Herd C, et al Functional profiling of a *Plasmodium* genome reveals an abundance of essential genes. Cell. 2017;170: 260–272. doi: 10.1016/j.cell.2017.06.030 28708996 10.1016/j.cell.2017.06.030 PMC5509546

[51] Cromer D, Evans KJ, Schofield L, Davenport MP. Preferential invasion of reticulocytes during late-stage *Plasmodium berghei* infection accounts for reduced circulating reticulocyte levels. Int J Parasitol. 2006;36: 1389–1397. doi: 10.1016/j.ijpara.2006.07.009 16979643 10.1016/j.ijpara.2006.07.009

[52] Martín-Jaular L, Elizalde-Torrent A, Thomson-Luque R, Ferrer M, Segovia JC, Herreros-Aviles E, et al Reticulocyte-prone malaria parasites predominantly invade CD71^hi^ immature cells: implications for the development of an *in vitro* culture for *Plasmodium vivax*. Malar J. 2013;12: 434 doi: 10.1186/1475-2875-12-434 24289105 10.1186/1475-2875-12-434 PMC4220676

[53] Srivastava A, Creek DJ, Evans KJ, De Souza D, Schofield L, Müller S, et al Host reticulocytes provide metabolic reservoirs that can be exploited by malaria parasites. PLoS Pathog. 2015;11: e1004882 doi: 10.1371/journal.ppat.1004882 26042734 10.1371/journal.ppat.1004882 PMC4456406

[54] Lehane AM, Marchetti RV, Spry C, van Schalkwyk DA, Teng R, Kirk K, et al Feedback inhibition of pantothenate kinase regulates pantothenol uptake by the malaria parasite. J Biol Chem. 2007;282: 25395–25405. doi: 10.1074/jbc.M704610200 17581817 10.1074/jbc.M704610200

[55] van der Westhuyzen R, Hammons JC, Meier JL, Dahesh S, Moolman WJA, Pelly SC, et al The antibiotic CJ-15,801 is an antimetabolite that hijacks and then inhibits CoA biosynthesis. Chem Biol. 2012;19: 559–571. doi: 10.1016/j.chembiol.2012.03.013 22633408 10.1016/j.chembiol.2012.03.013 PMC3361698

[56] Hong BS, Yun MK, Zhang Y-M, Chohnan S, Rock CO, White SW, et al Prokaryotic type II and type III pantothenate kinases: The same monomer fold creates dimers with distinct catalytic properties. Structure. 2006;14: 1251–1261. doi: 10.1016/j.str.2006.06.008 16905099 10.1016/j.str.2006.06.008

[57] Kumar P, Chhibber M, Surolia A. How pantothenol intervenes in Coenzyme-A biosynthesis of *Mycobacterium tuberculosis*. Biochem Biophys Res Commun. 2007;361: 903–909. doi: 10.1016/j.bbrc.2007.07.080 17679145 10.1016/j.bbrc.2007.07.080

[58] Zhang Y-M, Frank MW, Virga KG, Lee RE, Rock CO, Jackowski S. Acyl carrier protein is a cellular target for the antibacterial action of the pantothenamide class of pantothenate antimetabolites. J Biol Chem. 2004;279: 50969–50975. doi: 10.1074/jbc.M409607200 15459190 10.1074/jbc.M409607200

[59] Leonardi R, Chohnan S, Zhang Y-M, Virga KG, Lee RE, Rock CO, et al A pantothenate kinase from *Staphylococcus aureus* refractory to feedback regulation by coenzyme A. J Biol Chem. 2005;280: 3314–3322. doi: 10.1074/jbc.M411608200 15548531 10.1074/jbc.M411608200

