# Supplementary material for: Mutations in the pantothenate kinase of Plasmodium falciparum confer diverse sensitivity profiles to antiplasmodial pantothenate analogues
Source: PLoS Pathog. 2018 Apr 3;14(4):e1006918. doi: 10.1371/journal.ppat.1006918 (PMC5882169; doi:10.1371/journal.ppat.1006918)
Supplement: S1 Materials and methods — (DOCX) [file ppat.1006918.s001.docx]

**Supporting Information - Materials and Methods**

**Plasmid preparation and nucleic acid purification**

The *Pfpank1*-pGlux-1 plasmid consists of the wild-type *Pfpank1*-coding sequence inserted within multiple cloning site III of pGlux-1, which contains the human dihydrofolate reductase (*hdhfr*) gene that confers resistance to WR99210 as a positive selectable marker. This places *Pfpank1* under the regulation of the *Plasmodium falciparum* chloroquine resistance transporter (*Pfcrt*) promoter, and upstream of the GFP-coding sequence. This plasmid construct allows the parasite to express a GFP-tagged *Pf*PanK1 and was used to localise the protein within the parasite. The *Pfpank1*-stop-pGlux-1 plasmid is identical to the *Pfpank1*-pGlux-1 construct but contains two stop codons between the *Pfpank1*-specific sequence and the GFP-coding sequence. This enables the parasite to express wild-type *Pf*PanK1 that is not GFP-tagged and was used to complement the mutant lines with wild-type *Pf*PanK1. The Δ*Pfpank1*-pCC-1 construct was generated using the pCC-1 plasmid [1] as the backbone (**Figure S2 a**). This plasmid contains the *hdhfr* gene cassette as a positive selectable marker. Two *Pfpank1*-specific sequences were cloned into sites flanking the *hdhfr* cassette to allow for homologous integration into the native *Pfpank1* locus of the transfected parasite. The plasmid also contains the *Saccharomyces cerevisiae* cytosine deaminase/phosphoribosyl transferase (*Scfcu*) gene that confers sensitivity to 5-fluorocytosine (5-FC) as a negative selectable marker. Parasites that integrate the *hdhfr* gene cassette into *Pfpank1* by homologous recombination and no longer carry the Δ*Pfpank1*-pCC-1 construct as an episome should lose the *Scfcu* gene and therefore be resistant to 5-FC, while parasites that maintain the episomal Δ*Pfpank*1-pCC-1 would be killed due to the toxic metabolite generated in the presence of *Scfcu*. The genomic regions for homologous integration were selected such that if the construct is successfully integrated into the *P. falciparum* genome by double crossover homologous recombination, the *hdhfr* gene cassette will replace a 500 base-pair region of the first exon of *Pfpank1*, and hence will disrupt the gene.

The *Pfpank1* sequence used to generate the *Pfpank1*-pGlux-1 and *Pfpank1*-stop-pGlux-1 plasmids was amplified from parasite RNA. The RNeasy Mini Kit (QIAGEN) was used to purify total RNA from saponin-isolated *P. falciparum* parasites (typically 2 $\times$ 10^7^ cells) according to the manufacturer’s protocol for purifying total RNA from animal cells. An optional 15 min DNase I incubation was included in the procedure to eliminate residual genomic DNA (gDNA). Complementary DNA (cDNA) was synthesised from total RNA using SuperScript II Reverse Transcriptase (ThermoFisher), with Oligo(dT)_12-18_ primer, and included an optional incubation with RNaseOUT Recombinant Ribonuclease Inhibitor, as per manufacturer’s instructions. When required, parasite gDNA was extracted from saponin-isolated trophozoite-stage parasites using a DNeasy Plant Mini Kit (QIAGEN) according to the manufacturer’s instructions. For quantitative polymerase chain reaction (qPCR), parasite gDNA was extracted from frozen (−80 °C) parasite infected-erythrocyte pellets using a QIAamp DNA Blood Midi Kit (QIAGEN) following the manufacturer’s instructions, and included an additional RNAse A digestion (1 mg/mL final concentration). When necessary, an additional phenol-chloroform extraction step was performed after gDNA extraction as follows: equal volumes (250 $\mu$L each) of phenol and chloroform were added to each sample and gently vortexed to mix. Samples were centrifuged (16,300 $\times$ *g*, 5 min) to separate the different phases. The aqueous phase was extracted and any residual transition phase removed by another centrifugation (16,300 $\times$ *g*, 5 min). The purified gDNA was then precipitated overnight (2.5 $\times$ volumes of absolute ethanol, 0.3 M sodium acetate, pH 5.2, −20 °C), washed once with ice-cold 70% ethanol and resuspended in 100 $\mu$L of 10 mM Tris, 0.5 mM ethylenediaminetetraacetic acid (EDTA), pH 9.0.

*Pfpank1*-specific sequences were subsequently amplified from cDNA (to generate the *Pfpank1*-pGlux-1 and *Pfpank1*-stop-pGlux-1 constructs) or gDNA of wild-type 3D7 strain *P. falciparum* (to generate the Δ*Pfpank1*-pCC-1 construct) using either KOD Hot Start DNA polymerase (Merck Millipore) or Platinum *Pfx* DNA polymerase (ThermoFisher), with the oligonucleotide primers listed in **Table S4**, following each manufacturer’s protocol. To generate the *Pfpank1*-stop-pGlux-1 and *Pfpank1*-pGlux-1 constructs, the In-Fusion cloning (Clontech) method was employed to insert the *Pfpank1*-coding sequence into pGlux-1, with and without the introduction of stop codons prior to the GFP-coding sequence, respectively. Before the cloning step, the pGlux-1 plasmid was linearised by sequential digestions with *Xho*I (ThermoFisher) and then *Kpn*I (New England Biolabs), according to each manufacturer’s recommendation. The In-Fusion reactions were set up with either the In-Fusion Dry-Down PCR Cloning Kit (for *Pfpank1*-pGlux-1) or the In-Fusion HD EcoDry Cloning Kit (for *Pfpank1*-stop-pGlux-1) essentially as described in the manufacturer’s protocol. To generate the Δ*Pfpank1*-pCC-1 construct, the *Pfpank1* flank fragments (amplified from parasite gDNA to contain specific restriction sites; **Table S4**) were digested with their respective restriction enzymes (*Sac*II and *Spe*I (both New England Biolabs) for the 5’ *Pfpank1* flank, and *Eco*RI (ThermoFisher) and *Avr*II (New England Biolabs) for the 3’ *Pfpank1* flank) according to each manufacturer’s protocol. Subsequently, the digested 5’ *Pfpank1* flank was ligated into a linearised pCC-1 plasmid (following *Sac*II and *Spe*I digestion performed as before) using a Quick T4 DNA ligase (New England Biolabs), following manufacturer’s protocol, to generate an intermediate plasmid. This intermediate plasmid was then linearised by *Eco*RI and *Avr*II digestion performed as above, and was ligated with the 3’ *Pfpank1* flank as described above to generate the final Δ*Pfpank1*-pCC-1 plasmid (**Figure S2 a**).

The purity and concentration of all DNA and RNA samples was determined using a NanoDrop ND-1000 spectrophotometer (Nanodrop Technologies). Sanger sequencing of DNA samples was performed at the Australian Genome Research Facility (Sydney or Brisbane) to confirm the accuracy of DNA sequences.

**SYBR Safe-based parasite proliferation assay**

The *in vitro* inhibition experiments carried out in this study were performed as described previously [2]. Briefly, synchronous ring-stage parasite-infected erythrocytes were suspended within wells of a 96-well plate, in a final volume of 200 $\mu$L complete medium (0.5% parasitaemia and 1% haematocrit) in the presence of the test compound dissolved in water or DMSO, with the highest concentration of DMSO not exceeding 0.1% v/v. Parasite-infected erythrocytes incubated in 500 nM chloroquine were used as a no-proliferation control, while those incubated in the absence of inhibitory compounds were used as a 100% parasite proliferation control. The parasite suspensions were then incubated at 37 °C for 96 h under a low-oxygen atmosphere (96% nitrogen, 3% carbon dioxide and 1% oxygen). At the end of the incubation, each 96-well plate was frozen at −20 °C (to lyse the cells) and thawed before being processed as follows: 100 $\mu$L was aliquoted from the content of the wells of each test plate into the corresponding wells of a new 96-well plate. Subsequently, 100 $\mu$L of lysis buffer (20 mM Tris, pH 7.5, 5 mM EDTA, 0.008% w/v saponin and 0.08% v/v Triton X-100) containing SYBR Safe DNA gel stain (1$\times$ final concentration in 200 $\mu$L) was added to each well. The SYBR Safe fluorescence signal in each well was then measured with a FLUOstar OPTIMA plate-reader with 490 nm excitation and 520 nm emission wavelengths, after setting the gain on a 100% parasite proliferation control well.

The *in vitro* pantothenate requirement experiments were performed as described above with the following modifications. All solutions were prepared in complete pantothenate-free medium and cells used for these experiments were washed twice in complete pantothenate-free medium (10 mL, 1,500 $\times$ *g* for 5 min) to remove any pantothenate. Ring stage-infected erythrocyte suspensions were then placed in the final suspension containing the desired concentration of pantothenate (pantothenate-free complete RPMI 1640 medium supplemented with 2-fold serial dilutions of pantothenate). In these experiments, parasite-infected erythrocytes incubated in the absence of pantothenate served as a no-proliferation control, while those incubated in 1 $\mu$M pantothenate (the pantothenate concentration normally present in complete medium used for *in vitro* parasite culture) served as a 100% parasite proliferation control.

Each data set was fitted with sigmoidal curves (typically either $y=y0+\frac{ax^{b}}{c^{b}+x^{b}}$ or $y=\frac{ax^{b}}{c^{b}+x^{b}}$, where $y$ represents percentage parasite proliferation, $y$0 represents minimum percentage parasite proliferation, $a$ represents the range of percentage parasite proliferation, $b$ represents the Hill coefficient, $c$ represents the IC_50_ or SC_50_ value and $x$ represents compound concentration) using a nonlinear least squares regression. The IC_50_ and SC_50_ values for these experiments were then determined from these sigmoidal curves and averaged from several independent experiments. The fold change, relative to the Parent line, in the sensitivity of each mutant clone to a particular compound was calculated as the ratio between the IC_50_ value obtained for the mutant clone from a single experiment and the average Parent IC_50_ value. The same was done for the corresponding *Pf*PanK1-complemented lines. IC_35_ values (obtained by the same method described above) were used to determine the CJ-15,801 fold change for PanOH-B and CJ-A because ≥ 50% inhibition of growth was not obtained for all of the experiments performed for these clones.

**Drug pressuring**

Parasite drug-pressuring cultures with PanOH and CJ-15,801 were initiated by exposing synchronous ring-stage Parent line parasites (10 mL culture of 2 or 4% parasitaemia and 2% haematocrit) to either analogue at the IC_50_ values obtained for the Parent line at the time (PanOH $\boldsymbol{=}$ 400 $\mu$M and CJ-15,801 $\boldsymbol{=}$ 75 $\mu$M). Parasites were then exposed to cycles of increasing drug-pressure that lasts about 2 – 4 weeks each. During each cycle, each culture was maintained continuously in the presence of a fixed concentration of the pressuring analogue, with a medium replacement every 48 h and fresh erythrocytes (100 $\mu$L) added at least once a week. Whenever the parasitaemia for each culture reached approximately 5 – 10%, a parasite proliferation assay was performed with predominantly ring-stage parasites as described above. If the IC_50_ value obtained for the assay was equal to or greater than the pressuring concentration, aliquots of the culture were cryopreserved, and the concentration of the pantothenate analogue was increased. The cycle was then repeated, each time increasing the compound concentration in a step-wise fashion (multiples of 2 from the initial pressuring concentration) until the pressured parasites became approximately 8$\times$ less sensitive than the Parent line to the selecting compounds. This process took 11 – 13 weeks. The drug-resistant parasites were subsequently cultured and cloned in the absence of pantothenate analogue pressure for at least 3 months. The clones were then confirmed to have retained resistance to the pantothenate analogue used in the selection process, and were subsequently maintained in a drug-free environment for the remainder of the study.

**Confocal microscopy**

To prepare coverslip-bound cells, parasite-infected erythrocytes (5 − 10% parasitaemia) were washed once (centrifuged at 500 $\times$ *g*, 5 min) and resuspended at approximately 2% haematocrit in phosphate buffered saline (PBS), before 1 − 2 mL was added to a polyethylenimine (PEI)-coated coverslip placed within a well of a 6-well plate. Plates were incubated with shaking for 15 min at room temperature, after which unbound cells were washed off the coverslips with PBS (2 mL per well, with a 2 min shaking incubation followed by aspiration).

For imaging of fixed cells, unstained coverslip-bound cells were fixed in 1 mL of PBS containing 4% (w/v) paraformaldehyde (Electron Microscopy Services) and 0.0075% (w/v) gluteraldehyde for 30 min at room temperature without shaking. The fixative was then removed and the coverslips washed in PBS three times as described above, before they were rinsed in water and dried. A drop of SLOWFADE (Invitrogen) containing the nuclear stain 4’,6-diamino-2-phenylindole (DAPI) was added to the centre of the coverslip, which was then inverted onto a microscope slide and sealed with nail polish, before being used for imaging.

Live cells were stained with Hoechst 33258 nuclear stain and bound to a coverslip for imaging. Briefly, parasite-infected erythrocytes were prepared in PBS (~4% haematocrit) as described above, pelleted (2,000 $\times$ *g*, 30 s), and resuspended in PBS containing 20 $\mu$g/mL Hoechst 33258. Cells were incubated in the dye for 20 min at room temperature with shaking, pelleted and washed three times with PBS (2,000 $\times$ *g*, 30 s), before being resuspended in PBS (~2% haematocrit) and bound to PEI-coated coverslips as described above. Shortly prior to imaging, 5 − 10 $\mu$L of PBS was added to the centre of the coverslip, before it was inverted onto a microscope slide and sealed with nail polish.

**Attempted disruption of *Pfpank1***

The *Pfpank1* disruption plasmid, $\Delta$*Pfpank1*-pCC-1, was transfected into wild-type 3D7 strain *P.* *falciparum*, and positive transfectants were selected as described previously [3].

WR99210-resistant $\Delta$*Pfpank1*-pCC-1-transfectant parasites were subsequently maintained in the absence of WR99210 for three weeks to promote the loss of episomal copies of the construct. WR99210 (10 nM) was re-introduced to predominantly ring-stage Δ*Pfpank1*-pCC-1-transfectant cultures. Complete medium and WR99210 were replaced daily after the re-introduction of WR99210, and fresh erythrocytes added when appropriate. Parasites from this culture (1 cycle off and on WR99210) were then either subjected to negative selection or maintained in the absence of WR99210 for a second three-week period to promote further loss of episomal copies of the Δ*Pfpank1*-pCC-1 construct. The second WR99210 cycle was carried out exactly as the first.

Negative selection was performed to isolate parasites in which the *hdhfr* selection cassette of the Δ*Pfpank1*-pCC-1 construct had been integrated into the genome by double crossover homologous recombination. In these parasites, the *Scfcu* gene that confers sensitivity to 5-FC should have been lost. Negative selection was initiated with parasites that had been through either 1 or 2 rounds of WR99210 cycling. 5-FC (232 nM) was introduced to cultures of predominantly trophozoite-stage parasites, while selection with WR99210 (10 nM) was maintained and the medium was replaced daily. When parasites were no longer observable in Giemsa-stained culture smears, the medium (along with WR99210 and 5-FC) was replaced every second day and fresh erythrocytes (100 $\mu$L) were added once a week. Once WR99210- and 5-FC-resistant parasites were observed in culture, the medium (containing WR99210 and 5-FC) was replaced daily, with fresh erythrocytes added as appropriate.

**[^14^C]Pantothenate phosphorylation by parasite lysate**

In the [^14^C]pantothenate phosphorylation time course, each reaction contained a final concentration of 50 mM Tris-HCl (pH 7.4), 5 mM ATP, 5 mM MgCl_2_ and 2 $\mu$M (0.1 $\mu$Ci/mL) [^14^C]pantothenate (American Radiolabeled Chemicals). The phosphorylation reactions were initiated by the addition of parasite lysate (at a concentration corresponding to ~2.0 $\times$ 10^7^ cells/mL) and were maintained at 37 °C throughout the experiment. For each condition, a reaction with identical components to the corresponding test reaction but lacking parasite lysate was prepared and this served as the negative (no phosphorylation) control. Reactions were terminated by transferring 50 $\mu$L aliquots (in duplicate) into wells of a 96-well white polystyrene microplate with either 0.45 $\mu$m polypropylene filter bottom wells and short drip directors (Whatman), or 0.2 $\mu$m hydrophilic PVDF membrane filter bottom wells (Corning), which had been pre-loaded with 50 $\mu$L 150 mM barium hydroxide. To determine total radioactivity, 50 $\mu$L aliquots of each phosphorylation reaction were transferred, in duplicate, and mixed thoroughly with 150 $\mu$L Microscint-40 by pipetting the mixture at least 50 times in the wells of an OptiPlate-96 microplate (Perkin-Elmer). All of these samples were then processed, measured and analysed as described previously [4].

Activity profiles of the *Pf*PanK enzymes from the different mutant clones and the Parent line were determined across the following ranges of pantothenate concentrations, with the range of [^14^C]pantothenate concentrations in brackets: 0.1 – 2 $\mu$M (0.005 – 0.1 $\mu$Ci/mL) for the Parent, 3.125 – 200 $\mu$M (0.1 $\mu$Ci/mL) for PanOH-A and (0.156 – 0.313 $\mu$Ci/mL) PanOH-B, and 6.25 – 800 $\mu$M (0.3 – 0.313 $\mu$Ci/mL) for CJ-A. The pantothenate phosphorylation reactions in these kinetic experiments were set up as described previously [4], with the following minor modifications. Each mL of the reactions contained lysates prepared from ~1.0 $\times$ 10^8^ cells. All of the reactions were designed such that the amount of pantothenate phosphorylated by the lysate increased linearly with time during the experiment (15 – 40 min for Parent, 13 – 90 min for PanOH-A, 0.5 – 4.2 h for PanOH-B and 1 – 28 h for CJ-A). The initial rate of each reaction was determined from the gradient of its individual linear regression. These rates were then plotted as a function of pantothenate concentration, and the Michaelis-Menten equation “$y=\frac{ax}{b+x}$” was fitted to the data, where $y$ represents *V*_0_, $a$ represents *V*_max_, $b$ represents *K*_m_ and $x$ represents substrate concentration. The Michaelis constant (*K*_m_) and maximal velocity (*V*_max_) parameters of the *Pf*PanK variants were then determined from each fitted curve. The specificity constant of each *Pf*PanK variant was calculated by dividing its *V*_max_ value by the corresponding *K*_m_ value. The relative specificity constant was in turn calculated by dividing the specificity constant value obtained for each variant in each independent repeat to the average Parent specificity constant value.

Pantothenate phosphorylation experiments were also performed in the presence of antiplasmodial pantothenate analogues to evaluate what effects the different *Pf*PanK1 mutations have on the inhibitory activities of these analogues. The reactions (each containing lysates equivalent to 5.0 $\times$ 10^7^ cells/mL and 0.1 $\mu$Ci/mL of [^14^C]pantothenate) were set up essentially as described previously [4] and were incubated at 37 °C for 10 min (PanOH-A), 40 min (Parent), 2 h (PanOH-B) or 5 h (CJ-A), during which the amount of pantothenate phosphorylated increased linearly with time in the absence of analogue. The pantothenate analogues were dissolved in water or DMSO, with the highest concentration of DMSO not exceeding 0.1% v/v. The amount of pantothenate phosphorylated in each reaction was expressed as a percentage of total pantothenate phosphorylation (wells without any test analogue). The data was then plotted as a function of analogue concentration and a suitable sigmoidal curve (either $y=y0+\frac{ax^{b}}{c^{b}+x^{b}}$ or $y=\frac{ax^{b}}{c^{b}+x^{b}}$, where $y$ represents percentage pantothenate phosphorylation, $y$0 represents minimum percentage pantothenate phosphorylation, $a$ represents the range of percentage pantothenate phosphorylation, $b$ represents the Hill coefficient, $c$ represents the IC_50_ value and $x$ represents analogue concentration) fitted to determine the IC_50_ of each analogue.

**Metabolism of N5-trz-C1-Pan**

Cultures of predominantly trophozoite-stage *P. falciparum*-infected erythrocytes (Parent line) were first concentrated to $>$95% parasitaemia using magnet-activated cell sorting as described elsewhere [5]. Following a 1 h-recovery at 37 °C in complete medium and an atmosphere of 96% nitrogen, 3% carbon dioxide and 1% oxygen, N5-trz-C1-Pan (1 $\mu$M) or an equivalent volume of the solvent control (0.01% v/v DMSO) was added in triplicate or quadruplicate to suspensions of the *P. falciparum*-infected erythrocytes in complete medium. After a 4 h exposure to N5-trz-C1-Pan and/or DMSO at 37 °C (again under an atmosphere of 96% nitrogen, 3% carbon dioxide and 1% oxygen), 3.0 $\times$ 10^7^ cells from each sample were pelleted by centrifugation at 650 $\times$ *g* for 3 min. The supernatant was discarded and the cells washed in ice-cold culture medium (1 mL, 650 $\times$ *g*, 3 min, 4 °C) followed by ice-cold PBS (500 $\mu$L, 650 $\times$ *g*, 3 min, 4 °C). To extract metabolites, the washed cells were resuspended in ice-cold methanol containing internal standards (1 $\mu$M Tris and 1 $\mu$M CHAPS) and incubated on a vortex mixer for 1 h at 4 °C. The samples were clarified by centrifugation (at 17,000 $\times$ *g*, 10 min, 4 °C) before being processed for liquid chromatography-mass spectrometry (LC-MS) analysis.

Metabolite samples were analysed by LC-MS as follows. Samples (10 $\mu$L) were separated over a ZIC-pHILIC column (5 $\mu$m, 4.6 $\times$ 150 mm; Merck) using 20 mM ammonium carbonate and acetonitrile as the mobile phases. The Q-Exactive MS operated at a mass resolution of 35,000 from *m/z* 85 to 1,050 and was fitted with a heated electrospray source that switched between positive and negative ionisation modes. Pooled technical replicates were analysed using data-dependent tandem mass spectrometry (MS/MS) to confirm the identity of unique features in downstream analysis.

**Bibliography**

1. Maier AG, Rug M, O'Neill MT, Brown M, Chakravorty S, Szestak T, et al. Exported proteins required for virulence and rigidity of *Plasmodium falciparum*-infected human erythrocytes. Cell. 2008;134: 48–61.

2. Spry C, Macuamule C, Lin Z, Virga KG, Lee RE, Strauss E, et al. Pantothenamides are potent, on-target inhibitors of *Plasmodium falciparum* growth when serum pantetheinase is inactivated. PLoS One. 2013;8: e54974.

3. Tjhin ET, Staines HM, van Schalkwyk DA, Krishna S, Saliba KJ. Studies with the *Plasmodium falciparum* hexokinase reveal that PfHT limits the rate of glucose entry into glycolysis. FEBS Lett. 2013;587: 3182–3187.

4. Spry C, Saliba KJ, Strauss E. A miniaturized assay for measuring small molecule phosphorylation in the presence of complex matrices. Anal Biochem. 2014;451: 76–78.

5. Spry C, Saliba KJ. The human malaria parasite *Plasmodium falciparum* is not dependent on host coenzyme A biosynthesis. J Biol Chem. 2009;284: 24904–24913.
