# Supplementary material for: Mutations in the pantothenate kinase of Plasmodium falciparum confer diverse sensitivity profiles to antiplasmodial pantothenate analogues
Source: PLoS Pathog. 2018 Apr 3;14(4):e1006918. doi: 10.1371/journal.ppat.1006918 (PMC5882169; doi:10.1371/journal.ppat.1006918)
Supplement: S1 Table — MCS: Multiple Cloning Site. (DOCX) [file ppat.1006918.s002.docx]

| **Primer Name**  **Primer Sequence (5’-3’)** | **Function** |
| --- | --- |
| *Pfpank1*-pGlux-1-5’primer **TTACATATAACTCGAG**ATGAGAAAGTATAAAAACG | PCR amplification of *Pfpank1* flanked by a 16-base sequence homologous with the linearisation site of pGlux-1 (in bold) that includes the *Xho*I restriction site (underlined). The transcription start codon is highlighted in blue. |
| *Pfpank1*-pGlux-1-3’primer **TCTTCTCCTTTACTGGTACC**GGCAAGGAAAAAACATCCAAGAGC | PCR amplification of *Pfpank1* flanked by a 20-base sequence homologous with the linearisation site of pGlux-1 (in bold) that includes the *Kpn*I restriction site (underlined). |
| 5’*Pfpank1*flank-5’primer GACTCCGCGGTAAACCTTTTTATTCATACG | PCR amplification of a *Sac*II/*Spe*I restriction site-flanked 5’ *Pfpank1* flank that includes the *Sac*II restriction site (underlined). |
| 5’*Pfpank1*flank-3’primer GATCACTAGTTTCATCATACAGATATTTCG | PCR amplification of a *Sac*II/*Spe*I restriction site-flanked 5’ *Pfpank1* flank that includes the *Spe*I restriction site (underlined). |
| 3’*Pfpank1*flank-5’primer GATCGAATTCAGCTATTGGGGGAGGCACC | PCR amplification of an *Eco*RI/*Avr*II restriction site-flanked 3’ *Pfpank1* flank that includes the *Eco*RI restriction site (underlined). |
| 3’*Pfpank1*flank-3’primer GACTCCTAGGAAAGATAACTATTCATGTCC | PCR amplification of an *Eco*RI/*Avr*II restriction site-flanked 3’ *Pfpank1* flank that includes the *Avr*II restriction site (underlined). |
| pCC-1MCS1-5’primer TGGAATACTAAATATATATCCAATGGCCCCT | Sanger sequencing of pCC-1 clones with the 5’ *Pfpank1* flank inserted into MCS one. |
| pCC-1MCS1-3’primer CCAATAGATAAAATTTGTAG | Sanger sequencing of pCC-1 clones with the 5’ *Pfpank1* flank inserted into MCS one. |
| pCC-1MCS2-5’primer CCTAATCATGTAAATCTTAAATTTTTC | Sanger sequencing of pCC-1 clones with the 3’ *Pfpank1* flank inserted into MCS two. |
| pCC-1MCS2-3’primer GTACTGAGAGTGCACCATATGCGG | Sanger sequencing of pCC-1 clones with the 3’ *Pfpank1* flank inserted into MCS two. |
| 5’*Pfpank1*flank-internal-3’primer1 ACGTACCTCCAATATCTAGCG | Sanger sequencing of pCC-1 clones with the 5’ *Pfpank1* flank inserted into MCS one. |
| 5’*Pfpank1*flank-internal-3’primer2 ATCCTTCTTCTCGAGGACATTCG | Sanger sequencing of pCC-1 clones with the 5’ *Pfpank1* flank inserted into MCS one. |
| 3’*Pfpank1*flank-internal-3’primer1 GAATTAAACCCTTGGCTACC | Sequencing of pCC-1 clones with the 3’ *Pfpank1* flank inserted into MCS two. |
| *Pfpank1*-internal-5’primer ATAACGTTAACAGGAGGAGG | Sanger sequencing of *Pfpank1*-pGlux-1 clones. |
| *Pfpank1*-internal-3’primer1 TGAATTAAACCCTTGGCTACC | Sanger sequencing of *Pfpank1*-pGlux-1 clones. |
| *Pfpank1*-internal-3’primer2 ACTCCATGTGTAAGTGATTCC | Sanger sequencing of *Pfpank1*-pGlux-1 clones. |
| *Pfpank1*-stop-pGlux-1-5’primer **TTACATATAACTCGAG**ATGAGAAAGTATAAAAACGAAT | PCR amplification of *Pfpank1* flanked by a 16-base sequence homologous with the linearisation site of pGlux-1 (in bold) that includes the *Xho*I restriction site (underlined). Blue: transcription start. |
| *Pfpank1*-stop-pGlux-1-3’primer **TCTTCTCCTTTACTGGTACC**CTACTAGGCAAGGAAAAAACA | PCR amplification of *Pfpank1* flanked by a 20-base sequence homologous with the linearisation site of pGlux-1 (in bold) that includes the *Kpn*I restriction site (underlined). Red: transcription stops. |
| *Pfpank1*-stop-internal-5’primer CAGGAGGAGGGGCACATA | Sanger sequencing of *Pfpank1*-stop-pGlux-1 clones. |
| *Pfpank1*-stop-internal-3’primer AGGATGGAAAGGAGAGGTTATT | Sanger sequencing of *Pfpank1*-stop-pGlux-1 clones. |
| *Pfpank1*-stop-external-3’primer GCATCACCTTCACCCTCTCC | Sanger sequencing of *Pfpank1*-stop-pGlux-1 clones. |
| *Pfpank1*-PvPanOHA-Wt-5’primer CCAAGTTTTATTTCCTAAGCACG | qPCR of Parent vs PanOH-A gDNA. Forward primer binds preferentially to the wild-type *Pfpank1* sequence at the site responsible for the D507N mutation in PanOH-A. |
| *Pfpank1*-PvPanOHA-Mut-5’primer CCAAGTTTTATTTCCTAAGCACA | qPCR of Parent vs PanOH-A gDNA. Forward primer binds preferentially to the mutant *Pfpank1* sequence at the site responsible for the D507N mutation in PanOH-A (red: discerning nucleotide). |
| *Pfpank1*-PvPanOHA-3’primer ATTTTGAATGATAAACTAGGCAAGG | qPCR of Parent vs PanOH-A gDNA. Common reverse primer. |
| *Pfpank1*-PvPanOHB-5’primer GACATATCTAAGTTAGATGACACTTTA | qPCR of Parent vs PanOH-B gDNA. Common forward primer. |
| *Pfpank1*-PvPanOHB-Wt-3’primer TATTTATGTGCCCCTCCTC | qPCR of Parent vs PanOH-B gDNA. Reverse primer binds preferentially to the wild-type *Pfpank1* sequence at the site responsible for the ∆G95 mutation in PanOH-B. |
| *Pfpank1*-PvPanOHB-Mut-3’primer TATTTATGTGCCCCTCCTG | qPCR of Parent vs PanOH-B gDNA. Reverse primer binds preferentially to the wild-type *Pfpank1* sequence at the site responsible for the ∆G95 mutation in PanOH-B (red: discerning nucleotide). |
| *Pfpank1*-PvCJA-Wt-5’primer AATAACGTTAACAGGAGGAGG | qPCR of Parent vs CJ-A gDNA. Forward primer binds preferentially to the wild-type *Pfpank1* sequence at the site responsible for the G95A mutation in CJ-A. |
| *Pfpank1*-PvCJA-Mut-5’primer AATAACGTTAACAGGAGGAGC | qPCR of Parent vs CJ-A gDNA. Forward primer binds preferentially to the mutant *Pfpank1* sequence at the site responsible for the G95A mutation in CJ-A (red: discerning nucleotide). |
| *Pfpank1*-PvCJA-3’primer GCCTTTATTTATTTCCATTCC | qPCR of Parent vs CJ-A gDNA. Common reverse primer. |
| *Pfpank1*-KO-determination-5’primer  TTACATATAACTCGAG**ATGAGAAAGTATAAAAACGAATTAAACA** | Determination of *Pfpank1* knockout when paired with *Pfpank1*-KO-determination-3’primer. A 1.9 kb product is expected when using template from wild-type parasites and a 3.6 kb product is expected with template from *Pfpank1* knockout parasites. Bold: sequence homologous with gene. |
| *Pfpank1*-KO-determination-3’primer  TCTTCTCCTTTACTGGTACC**CTACTAGGCAAGGAAAAAACATCC** | Determination of *Pfpank1* knockout when paired with *Pfpank1*-KO-determination-5’primer. A 1.9 kb product is expected when using template from wild-type parasites and a 3.6 kb product is expected with template from *Pfpank1* knockout parasites. Bold: sequence homologous with gene. |
