# Supplementary material for: Mutations in the pantothenate kinase of Plasmodium falciparum confer diverse sensitivity profiles to antiplasmodial pantothenate analogues
Source: PLoS Pathog. 2018 Apr 3;14(4):e1006918. doi: 10.1371/journal.ppat.1006918 (PMC5882169; doi:10.1371/journal.ppat.1006918)
Supplement: S2 Table — aCertainty of peptide identification. (DOCX) [file ppat.1006918.s003.docx]

| **Protein** | **Peptide**  **(start and end position, [+16] oxidation)** | **Score^a^** | ***m/z*** | **Mass**  **(Da)** | **Retention time (min)** |
| --- | --- | --- | --- | --- | --- |
| *Pf*PanK1 | 296'_IAGTAIGGGTLMGLAK_'311 | 95.07 | 715.9051 | 1429.796 | 78.2 |
| *Pf*PanK1 | 6'_NELNISNVLEK_'16 | 90.7 | 636.843 | 1271.672 | 72 |
| *PfP*anK2 | 257'_FMPTNLYYFTK_'267 | 64.2 | 712.849 | 1423.684 | 89.9 |
| *PfP*anK2 | 393'_DLTASFFGNAQHIENVK_'409 | 48.1 | 945.9696 | 1889.925 | 83.2 |
| *Pf*HSP70 | 172'_DAGTIAGLNVM[+16]R_'183 | 81.2 | 617.31643 | 1232.6183 | 61.1 |
| *Pf*HSP70 | 172'_DAGTIAGLNVMR_'283 | 75.8 | 609.31966 | 1216.6248 | 76.4 |
| *Pf*HSP70 | 325'_DTLIPVEK_'332 | 91.1 | 457.763 | 913.5115 | 57.4 |
| *Pf*HSP70 | 611'_EAESVCAPIMSK_'622 | 114.7 | 661.30978 | 1320.605 | 48.4 |
| *Pf*HSP70 | 573'_EKLQPAEIETCMK_'585 | 77.8 | 788.8898 | 1575.765 | 45.12 |
| *Pf*HSP70 | 141'_ENAEAFLGK_'149 | 79.15 | 489.7481 | 977.4817 | 49.6 |
| *Pf*HSP70 | 315'_FEELCIDYFR_'324 | 161.2 | 696.31842 | 1390.6223 | 93.4 |
| *Pf*HSP70 | 472'_FHLDGIPPAPR_'482 | 144.8 | 610.3331 | 1218.652 | 59.5 |
| *Pf*HSP70 | 472'_FHLDGIPPAPR_'482 | 89 | 407.2242 | 1218.651 | 58.8 |
| *Pf*HSP70 | 89'_FTESSVQSDM[+16]K_'99 | 103.4 | 610.3331 | 1218.652 | 25.2 |
| *Pf*HSP70 | 89'_FTESSVQSDMK_'99 | 85.8 | 629.7854 | 1257.556 | 38.4 |
| *Pf*HSP70 | 100'_HWPFTVK_'106 | 173.9 | 457.7479 | 913.481 | 53.6 |
| *Pf*HSP70 | 184'_IINEPTAAAIAYGLHK_'199 | 109.8 | 841.4695 | 1680.924 | 67.2 |
| *Pf*HSP70 | 184'_IINEPTAAAIAYGLHK_'199 | 47.5 | 561.3134 | 1680.918 | 69.1 |
| *Pf*HSP70 | 88'_KFTESSVQSDMK_'99 | 234.8 | 693.8322 | 1385.65 | 29.2 |
| *Pf*HSP70 | 88'_KFTESSVQSDMK_'99 | 88.1 | 462.8904 | 1385.649 | 29.2 |
| *Pf*HSP70 | 125'_LFHPEEISSM[+16]VLQK_'138 | 46.8 | 837.4352 | 1672.856 | 65.4 |
| *Pf*HSP70 | 125'_LFHPEEISSM[+16]VLQK_'138 | 59.3 | 558.6239 | 1672.85 | 64.7 |
| *Pf*HSP70 | 125'_LFHPEEISSMVLQK_'138 | 190.3 | 829.4345 | 1656.855 | 77.9 |
| *Pf*HSP70 | 125'_LFHPEEISSMVLQK_'138 | 136.01 | 553.2919 | 1656.854 | 77.89 |
| *Pf*HSP70 | 249'_LVNFCVEDFK_'258 | 113.9 | 635.81022 | 1269.6059 | 80.6 |
| *Pf*HSP70 | 249'_LVNFCVEDFKR_'259 | 121.4 | 713.86108 | 1425.7076 | 64.9 |
| *Pf*HSP70 | 139'_M[+16]KENAEAFLGK_'140 | 119.4 | 627.3124 | 1252.61 | 34.7 |
| *Pf*HSP70 | 139'_M[+16]KENAEAFLGK_'140 | 89.3 | 418.5446 | 1252.612 | 34.7 |
| *Pf*HSP70 | 139'_MKENAEAFLGK_'140 | 101.4 | 619.3164 | 1236.618 | 41.5 |
| *Pf*HSP70 | 139'_MKENAEAFLGK_'140 | 48.9 | 413.231 | 1236.617 | 41.4 |
| *Pf*HSP70 | 153'_NAVITVPAYFNDSQR_'167 | 159.2 | 847.92906 | 1693.8436 | 80.43 |
| *Pf*HSP70 | 34'_NENVDIIANDQGNR_'47 | 128.2 | 786.3726 | 1570.731 | 43 |
| *Pf*HSP70 | 73'_NPENTVFDAK_'82 | 125.7 | 567.775 | 1133.536 | 39.3 |
| *Pf*HSP70 | 73'_NPENTVFDAKR_'83 | 74.6 | 645.8253 | 1289.636 | 29.6 |
| *Pf*HSP70 | 553'_NSLENYCYGVK_'563 | 104.4 | 673.80534 | 1345.5961 | 49.1 |
| *Pf*HSP70 | 107'_SGVDEKPM[+16]IEVTYQGEK_'123 | 184.8 | 963.4614 | 1924.908 | 46.2 |
| *Pf*HSP70 | 107'_SGVDEKPM[+16]IEVTYQGEK_'123 | 138.9 | 642.6434 | 1924.908 | 46.2 |
| *Pf*HSP70 | 107'_SGVDEKPMIEVTYQGEK_'123 | 152.01 | 955.464 | 1908.913 | 53.9 |
| *Pf*HSP70 | 107'_SGVDEKPMIEVTYQGEK_'123 | 141.1 | 637.3119 | 1908.9 | 53.9 |
| *Pf*HSP70 | 107'_SGVDEKPMIEVTYQGEKK_'124 | 95.2 | 685.3418 | 2053.004 | 36.9 |
| *Pf*HSP70 | 438'_SQIFTTYADNQPGVLIQVYEGER_'460 | 57.2 | 876.7689 | 2627.285 | 102.5 |
| *Pf*HSP70 | 343'_SVHEVVLVGGSTR_'355 | 79.9 | 670.3708 | 1338.727 | 37.7 |
| *Pf*HSP70 | 343'_SVHEVVLVGGSTR_'355 | 79.7 | 447.2489 | 1338.725 | 37.7 |
| *Pf*HSP70 | 586'_TITTILEWLEK_'596 | 111.63 | 673.8821 | 1345.75 | 119.4 |
|  |  |  |  |  |  |
| Table continued on next page | | | | | |
|  |  |  |  |  |  |
|  |  |  |  |  |  |
| Table continued from previous page. | | | | | |
|  |  |  |  |  |  |
| *Pf*HK | 337'_FMVNVLQSACSK_'348 | 112.8 | 692.34118 | 1382.6678 | 72.7 |
| *Pf*HK | 429'_FTCGVDGSLFVK_'440 | 104.2 | 665.32943 | 1328.6443 | 74 |
| *Pf*HK | 475'_GAAITAAVIALNADIPQLP_'493 | 21.7 | 607.0155 | 1818.0247 | 136.7 |
| *Pf*HK | 475'_GAAITAAVIALNADIPQLP_'493 | 81.7 | 910.02112 | 1818.0277 | 136.7 |
| *Pf*HK | 96'_GTYYAIDFGGTNFR_'109 | 99.7 | 791.37023 | 1580.7259 | 85.8 |
| *Pf*HK | 398'_ICEAVYNR_'405 | 161.7 | 512.74789 | 1023.4812 | 31.5 |
| *Pf*HK | 288'_IINIEFGNFDK_'298 | 98.2 | 655.34292 | 1308.6713 | 87.6 |
| *Pf*HK | 221'_LMNDAFVR_'228 | 96.1 | 483.24716 | 964.47977 | 53.3 |
| *Pf*HK | 81'_M[+16]LDSCIANIPTGQEK_'95 | 142.2 | 846.90042 | 1691.7863 | 60.1 |
| *Pf*HK | 324'_MISGAYLGEIVR_'335 | 97.5 | 654.85283 | 1307.6911 | 80.6 |
| *Pf*HK | 81'_MLDSCIANIPTGQEK_'95 | 150.8 | 838.90303 | 1675.7915 | 66.7 |
| *Pf*HK | 406'_SAALAAGTIAAIAK_'419 | 127.8 | 614.86649 | 1227.7184 | 68.9 |
| *Pf*HK | 454'_VILADKAENLIIIPADDGSGK_'474 | 81.8 | 718.0673 | 2151.1801 | 85.1 |
| *Pf*HK | 162'_YIMGEFNDLDNK_'173 | 111.9 | 729.83211 | 1457.6497 | 74.2 |
| *Pf*α-tubulin | 44'_ANDDAFNTFFSETGAGK_'60 | 88.2 | 896.3956 | 1790.777 | 91.5 |
| *Pf*α-tubulin | 65'_CVFVDLEPTVVDEVR_'79 | 97.8 | 888.94593 | 1775.8773 | 96.8 |
| *Pf*α-tubulin | 327'_DVNAAVATIK_'336 | 90.3 | 501.28487 | 1000.5552 | 50 |
| *Pf*α-tubulin | 423'_EDLAALEK_'430 | 80.2 | 444.73726 | 887.45996 | 45.5 |
| *Pf*α-tubulin | 113'_EVIDVCLDR_'121 | 134.2 | 559.77937 | 1117.5442 | 64.02 |
| *Pf*α-tubulin | 244'_FDGALNVDVTEFQTNLVPYPR_'264 | 14.1 | 799.06999 | 2394.1881 | 112.9 |
| *Pf*α-tubulin | 395'_FDLMYAK_'401 | 99.2 | 444.22022 | 886.42589 | 65.9 |
| *Pf*α-tubulin | 265'_IHFMLSSYAPVVSAEK_'280 | 81.7 | 889.9615 | 1777.908 | 78.5 |
| *Pf*α-tubulin | 265'_IHFMLSSYAPVVSAEK_'280 | 89.9 | 593.6431 | 1777.908 | 78.6 |
| *Pf*α-tubulin | 230'_LIAQVISSLTASLR_'243 | 138.2 | 736.44601 | 1470.8775 | 119 |
| *Pf*α-tubulin | 216'_NLDIERPTYTNLNR_'229 | 156.2 | 573.63288 | 1717.8768 | 54.5 |
| *Pf*α-tubulin | 85'_QLFHPEQLISGK_'96 | 182.5 | 698.88302 | 1395.7515 | 64.5 |
