# Supplementary material for: Mutations in the pantothenate kinase of Plasmodium falciparum confer diverse sensitivity profiles to antiplasmodial pantothenate analogues
Source: PLoS Pathog. 2018 Apr 3;14(4):e1006918. doi: 10.1371/journal.ppat.1006918 (PMC5882169; doi:10.1371/journal.ppat.1006918)
Supplement: S3 Table — Errors represent SEM (n ≥ 3). The averaged CJ-15,801 IC50 value for PanOH-B (n = 3) includes a single extrapolated value, because in one experiment the highest concentration tested (800 μM) did not inhibit parasite growth by ≥ 50%. An asterisk indicates that the IC50 value of the mutant line is significantly different from that obtained for the Parent line (PanOH IC50 95% CI compared to Parent IC50: PanOH-A = 2664 to 3432, PanOH-B = 3553 to 4552 & CJ-A = 6046 to 7456; CJ-15,801 IC50 95% CI compared to Parent IC50: PanOH-A = 296 to 436, PanOH-B = 495 to 717 & CJ-A = 688 to 823). The chloroquine IC50 values of the different lines are indistinguishable (95% CI compared to Parent IC50: PanOH-A = -2.023 to 1.646, PanOH-B = -2.114 to 0.492 & CJ-A = -2.507 to 0.158). (DOCX) [file ppat.1006918.s004.docx]

| **Parasite line** | **Parasite proliferation inhibition IC_50_ values (**$\boldsymbol{\mu}$**M)** | | |
| --- | --- | --- | --- |
|  | **PanOH** | **CJ-15,801** | **Chloroquine** |
| **Parent (3D7 strain)** | 547 ± 35 | 106 ± 8 | 0.009 ± 0.001 |
| **PanOH-A** | 3,595 ± 338* | 471 ± 54* | 0.009 ± 0.001 |
| **PanOH-B** | 4,600 ± 453* | 712 ± 110* | 0.008 ± 0.001 |
| **CJ-A** | 7,298 ± 745* | >800 | 0.008 ± 0.001 |
| **Parent^+WT^*^Pf^*^PanK1^** | 672 ± 37 | 127 ± 7 | 0.010 ± 0.001 |
| **PanOH-A^+WT^*^Pf^*^PanK1^** | 1946 ± 222 | 252 ± 37 | 0.010 ± 0.001 |
| **PanOH-B^+WT^*^Pf^*^PanK1^** | 2786 ± 170 | 357 ± 23 | 0.010 ± 0.001 |
| **CJ-A^+WT^*^Pf^*^PanK1^** | 3036 ± 203 | 302 ± 29 | 0.011 ± 0.001 |
