# Supplementary material for: Mutations in the pantothenate kinase of Plasmodium falciparum confer diverse sensitivity profiles to antiplasmodial pantothenate analogues
Source: PLoS Pathog. 2018 Apr 3;14(4):e1006918. doi: 10.1371/journal.ppat.1006918 (PMC5882169; doi:10.1371/journal.ppat.1006918)
Supplement: S4 Table — Uppercase letters in the “Codon Change” column denotes the base within the codon that has been altered in the coding sequence before/after the mutation. The deletion in PanOH-B at position 95 is not included here because PlaTyPus is unable to detect insertions-deletions polymorphisms. (DOCX) [file ppat.1006918.s005.docx]

**List of non-synonymous mutations in PanOH-A**

| Chromosome | Position | Gene Name | Gene Description | Ref Base | Alt  Base | Codon  Change | Amino Acid  Change |
| --- | --- | --- | --- | --- | --- | --- | --- |
| Pf3D7_12_v3 | 283230 | PF3D7_1206300 | conserved protein,  unknown function | C | T | Gaa/Aaa | E2025K |
| Pf3D7_14_v3 | 854135 | PF3D7_1420600 | pantothenate kinase,  putative (PANK) | C | T | Gat/Aat | D507N |
| Pf3D7_08_v3 | 1080639 | PF3D7_0824900 | conserved protein,  unknown function | C | T | Gat/Aat | D785N |
| Pf3D7_11_v3 | 704658 | PF3D7_1118500 | nucleolar protein 56,  putative | G | A | aGa/aAa | R495K |

| Chromosome | Position | Gene Name | Gene Description | Ref Base | Alt  Base | Codon  Change | Amino Acid  Change |
| --- | --- | --- | --- | --- | --- | --- | --- |
| Pf3D7_12_v3 | 283209 | PF3D7_1206300 | conserved protein,  unknown function | C | T | Gaa/Aaa | E2032K |
| Pf3D7_01_v3 | 318038 | PF3D7_0107600 | serine/threonine  protein kinase, putative | A | C | Aat/Cat | N1141H |

**List of non-synonymous mutations in PanOH-B**

**List of non-synonymous mutations in CJ-A**

| Chromosome | Position | Gene Name | Gene Description | Ref  Base | Alt  Base | Codon  Change | Amino Acid  Change |
| --- | --- | --- | --- | --- | --- | --- | --- |
| Pf3D7_09_v3 | 762391 | exon |  | G | T | tGt/tTt | C394F |
| Pf3D7_14_v3 | 855680 | PF3D7_1420600 | pantothenate kinase, putative (PANK) | C | G | gGg/gCg | G95A |
| Pf3D7_10_v3 | 1493497 | PF3D7_1037600 | DNA repair helicase  RAD25, putative | A | G | Aaa/Gaa | K594E |
