# Supplementary material for: Mutations in the pantothenate kinase of Plasmodium falciparum confer diverse sensitivity profiles to antiplasmodial pantothenate analogues
Source: PLoS Pathog. 2018 Apr 3;14(4):e1006918. doi: 10.1371/journal.ppat.1006918 (PMC5882169; doi:10.1371/journal.ppat.1006918)
Supplement: S5 Table — Errors represent SEM (n ≥ 3). An asterisk indicates that the value is significantly different from that obtained for the Parent line (95% CI of N5-trz-C1-Pan proliferation inhibition IC50 compared to Parent: PanOH-A = -0.088 to -0.038, PanOH-B = 0.047 to 0.121 & CJ-A = 0.715 to 0.824; 95% CI of PE-αMe-PanAm proliferation inhibition IC50 compared to Parent: PanOH-A = -0.060 to -0.008 & CJ-A = 0.024 to 0.076; 95% CI of PanOH phosphorylation inhibition IC50 compared to Parent: PanOH-A = 46 to 69, PanOH-B = 71 to 263 & CJ-A = 4531 to 5300; 95% CI of CJ-15,801 phosphorylation inhibition IC50 compared to Parent: PanOH-A = 10 to 309 & PanOH-B = 330 to 621; 95% CI of N5-trz-C1-Pan phosphorylation inhibition IC50 compared to Parent: PanOH-A = 6.5 to 13 & PanOH-B = 9.8 to 30; 95% CI of N-PE-αMe-PanAm phosphorylation inhibition IC50 compared to Parent: PanOH-B = 3.6 to 9.0 & CJ-A = 75 to 97). (DOCX) [file ppat.1006918.s006.docx]

| **Parasite  line** |  | **Parasite proliferation inhibition  IC_50_ values (**$\boldsymbol{\mu}$**M)** | | | | |  | **Pantothenate phosphorylation inhibition  IC_50_ values (**$\boldsymbol{\mu}$**M)** | | | | |
| --- | --- | --- | --- | --- | --- | --- | --- | --- | --- | --- | --- | --- |
|  |  | **N5-trz- C1-Pan** | ***N*-PE-αMe-**  **PanAm** |  | | **PanOH** | | | | **CJ-15,801** | **N5-trz- C1-Pan** | ***N*-PE-αMe-**  **PanAm** |
| **Parent** |  | 0.095 ± 0.007 | 0.060 ± 0.007 |  | 9.6 ± 0.7 | | | | 37 ± 7 | | 1.7 ± 0.1 | 0.70 ± 0.13 |
| **PanOH-A** |  | 0.032 ± 0.001* | 0.026 ± 0.004* |  | 67 ± 4* | | | | 196 ± 53* | | 11 ± 1* | 1.4 ± 0.2 |
| **PanOH-B** |  | 0.179 ± 0.018* | 0.040 ± 0.004 |  | 176 ± 35* | | | | 512 ± 52* | | 22 ± 4* | 7 ± 1* |
| **CJ-A** |  | 0.864 ± 0.028* | 0.110 ± 0.004* |  | 4925 ± 138* | | | | >1600 | | >100 | 81 ± 6* |
