# Supplementary figures and images for: Mutations in the pantothenate kinase of Plasmodium falciparum confer diverse sensitivity profiles to antiplasmodial pantothenate analogues
Source: PLoS Pathog. 2018 Apr 3;14(4):e1006918. doi: 10.1371/journal.ppat.1006918 (PMC5882169; doi:10.1371/journal.ppat.1006918)

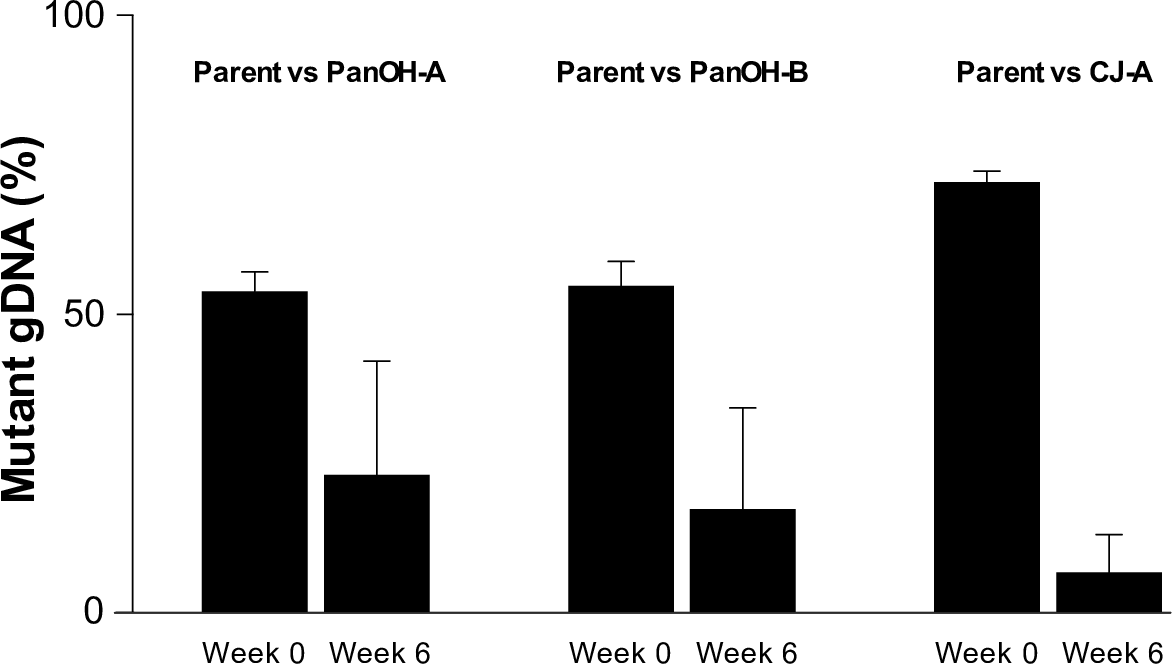

Supplement: S1 Fig — Data are shown as the proportions of mutant gDNA (out of a 100% total) at week 0 and week 6. Values are averaged from two independent competition assays, each analysed by qPCR in duplicate. Error bars represent range/2. (TIF) [file ppat.1006918.s007.tif]

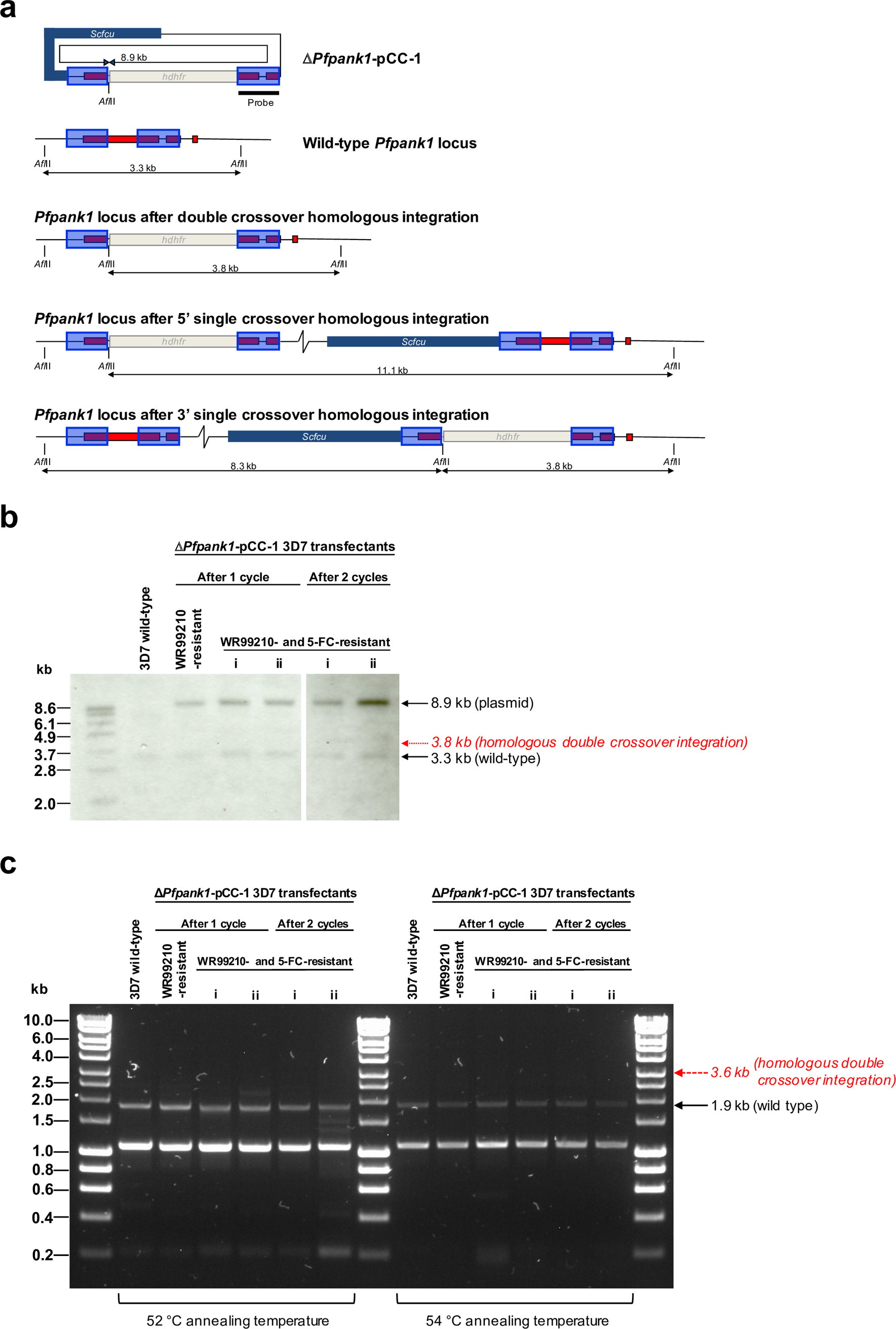

Supplement: S2 Fig — (a) Schematic representations of the ΔPfpank1-pCC-1 construct and the wild-type Pfpank1 gene locus before and after either a homologous double crossover integration of the hdhfr cassette of ΔPfpank1-pCC-1 or a 5’/3’ single crossover homologous integration of the ΔPfpank1-pCC-1 construct. The positions of AflII restriction sites are indicated. The 5’ Pfpank1 and 3’ Pfpank1 homologous flanks are indicated by translucent blue boxes. (b) Southern blot of AflII-digested gDNA extracted from wild-type parasites and from ΔPfpank1-pCC-1-transfectants resistant to WR99210 or both WR99210 and 5-fluorocytosine (5-FC) after one or two rounds of WR99210 cycling. (i) and (ii) represent independently-selected drug-resistant cultures. The blot was probed with the 3’ Pfpank1 flank (as indicated by the black bar in (a)). The probe hybridised to fragments that correspond to the 3D7 wild-type (3.3 kb) and the plasmid (8.9 kb). The fragment that is consistent with a homologous double crossover-disrupted locus (3.8 kb) was not detected in either independent culture. hdhfr: human dihydrofolate reductase—conveys resistance to WR99210. Scfcu: Saccharomyces cerevisiae cytosine deaminase/phosphoribosyl transferase—conveys sensitivity to 5-FC. (c) PCR confirmation that a Pfpank1 knockout event cannot be detected, even in a small sub-population of parasites. Samples from the same parasite populations shown in (b) were used to generate DNA templates that were then used with primers selected to amplify a product of 1.9 kb from the wild-type sequence and a 3.6 kb product from parasites with the Pfpank1 gene knocked out (and replaced with the hdhfr gene). A PCR product consistent with the size expected for amplification from the wild-type sequence was observed (black arrow). The identity of this product was confirmed by sequencing. All the reactions generated an approximately 1.1 kb PCR product and some reactions, under certain conditions, produced fainter PCR products likely due to non-specific pri [file ppat.1006918.s008.tif]

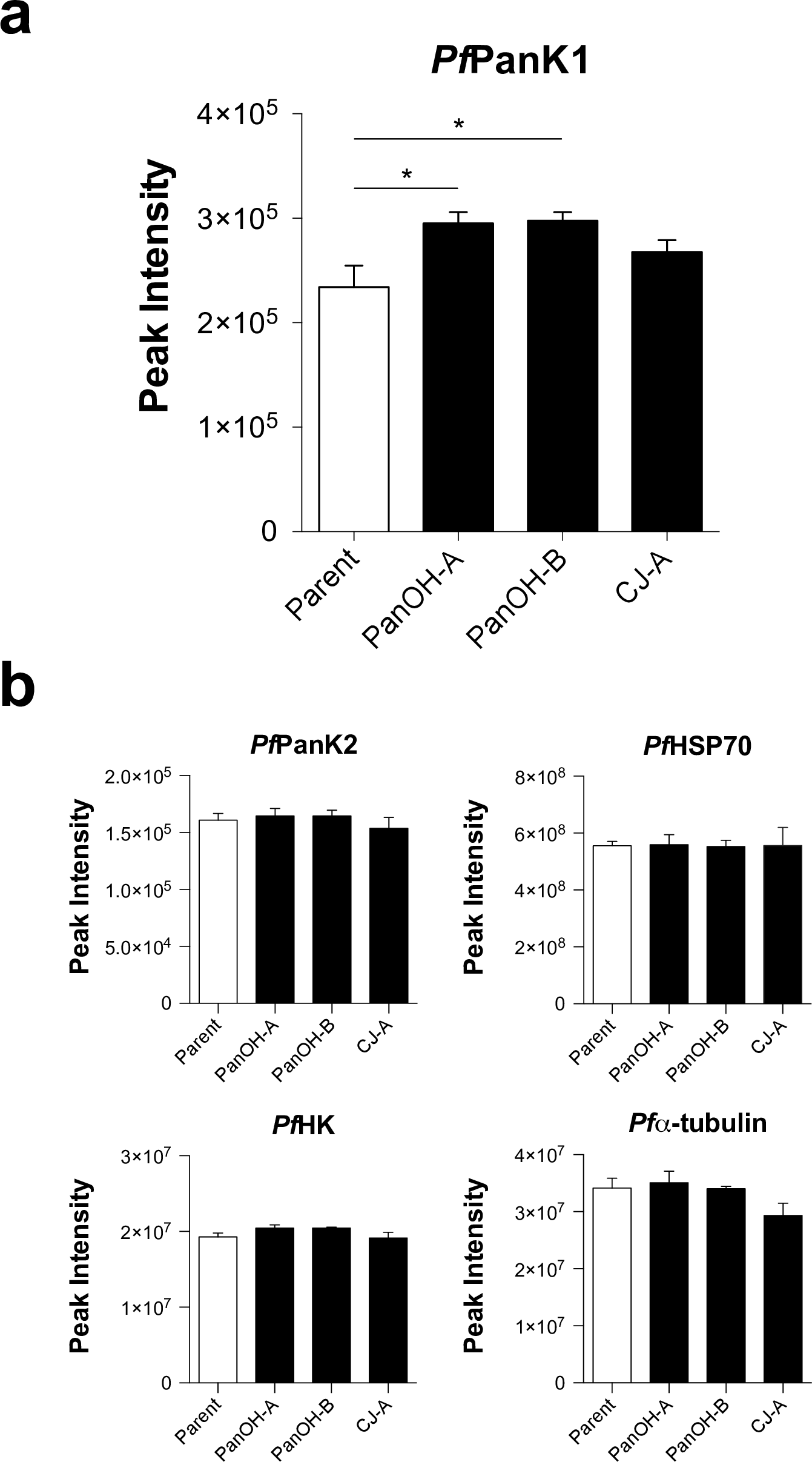

Supplement: S3 Fig — Abundance of (a) PfPanK1 and (b) various control proteins (PfPanK2, PfHSP70, PfHK and Pfα-tubulin) in Parent, PanOH-A, PanOH-B and CJ-A line trophozoites. Protein levels are determined by LC-MS/MS followed by DIA analysis. Values are averaged from ≥ 3 independent parasite preparations and error bars represent SEM. An asterisk indicates that the protein peak intensity measured for a mutant line is significantly different from that obtained for the Parent line (95% CI of PfPanK1 protein level compared to Parent: PanOH-A = 3.87 × 10^3^ to 1.18 × 10^5^ & PanOH-B = 8.86 × 10^3^ to 1.18 × 10^5^). The protein abundance of PfPanK1 in CJ-A (95% CI compared to Parent = -3.38 × 10^4^ to 1.01 × 10^5^) and those of the housekeeping proteins in all three mutant lines are indistinguishable from the Parent line levels (95% CI for PfPanK2 level compared to Parent: PanOH-A = -1.81 × 10^4^ to 2.53 × 10^4^, PanOH-B = -1.52 × 10^4^ to 2.25 × 10^4^ & CJ-A = -3.43 × 10^4^ to 1.99 × 10^4^; 95% CI for PfHSP70 level compared to Parent: PanOH-A = -8.98 × 10^7^ to 9.72 × 10^7^, PanOH-B = -6.92 × 10^7^ to 6.27 × 10^7^ & CJ-A = -1.45 × 10^8^ to 1.45 × 10^8^; 95% CI for PfHK level compared to Parent: PanOH-A = -4.41 × 10^5^ to 2.79 × 10^6^, PanOH-B = -1.18 × 10^5^ to 2.47 × 10^6^ & CJ-A = -2.42 × 10^6^ to 2.13 × 10^6^; 95% CI for Pfα-tubulin level compared to Parent: PanOH-A = -5.57 × 10^6^ to 7.45 × 10^6^, PanOH-B = -4.47 × 10^6^ to 4.25 × 10^6^ & CJ-A = -1.18 × 10^7^ to 2.22 × 10^6^). (TIF) [file ppat.1006918.s009.tif]

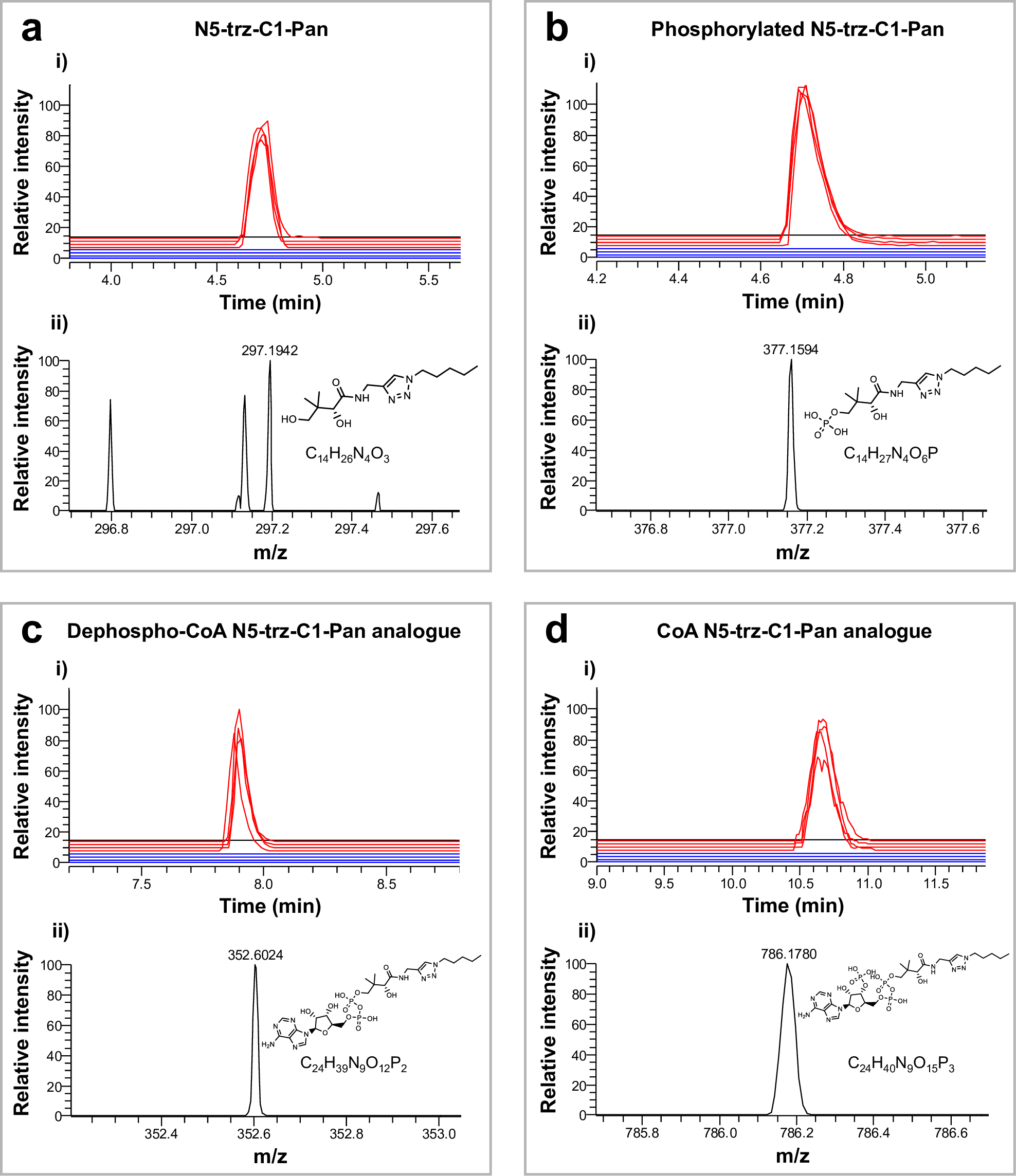

Supplement: S4 Fig — Extracted ion chromatograms (i) and mass spectra (ii) of N5-trz-C1-Pan (a) and downstream metabolites (b–d). Extracted ion chromatograms (i) show the relative intensity of each LC peak. Red lines represent metabolites extracted from N5-trz-C1-Pan-treated parasites and blue lines represent those extracted from DMSO-treated parasite control samples, each carried out in quadruplicate. High resolution mass spectra (ii) of each compound ionised in negative mode: (a) N5-trz-C1-Pan (C14H26N4O3). Theoretical m/z = 297.1932. Observed m/z = 297.1942. Δppm = 3.36. (b) Phosphorylated N5-trz-C1-Pan (C14H27N4O6P). Theoretical m/z = 377.1595. Observed m/z = 377.1594. Δppm = -0.27. (c) Dephospho-CoA N5-trz-C1-Pan analogue (C24H39N9O12P2). Theoretical m/z = 352.6024. Observed m/z = 352.6024. Δppm = 0.0. (d) CoA N5-trz-C1-Pan analogue (C24H40N9O15P3). Theoretical m/z = 786.1784. Observed m/z = 786.1780. Δppm = -0.51. Data shown are from a single experiment representative of two independent experiments. (TIF) [file ppat.1006918.s010.tif]

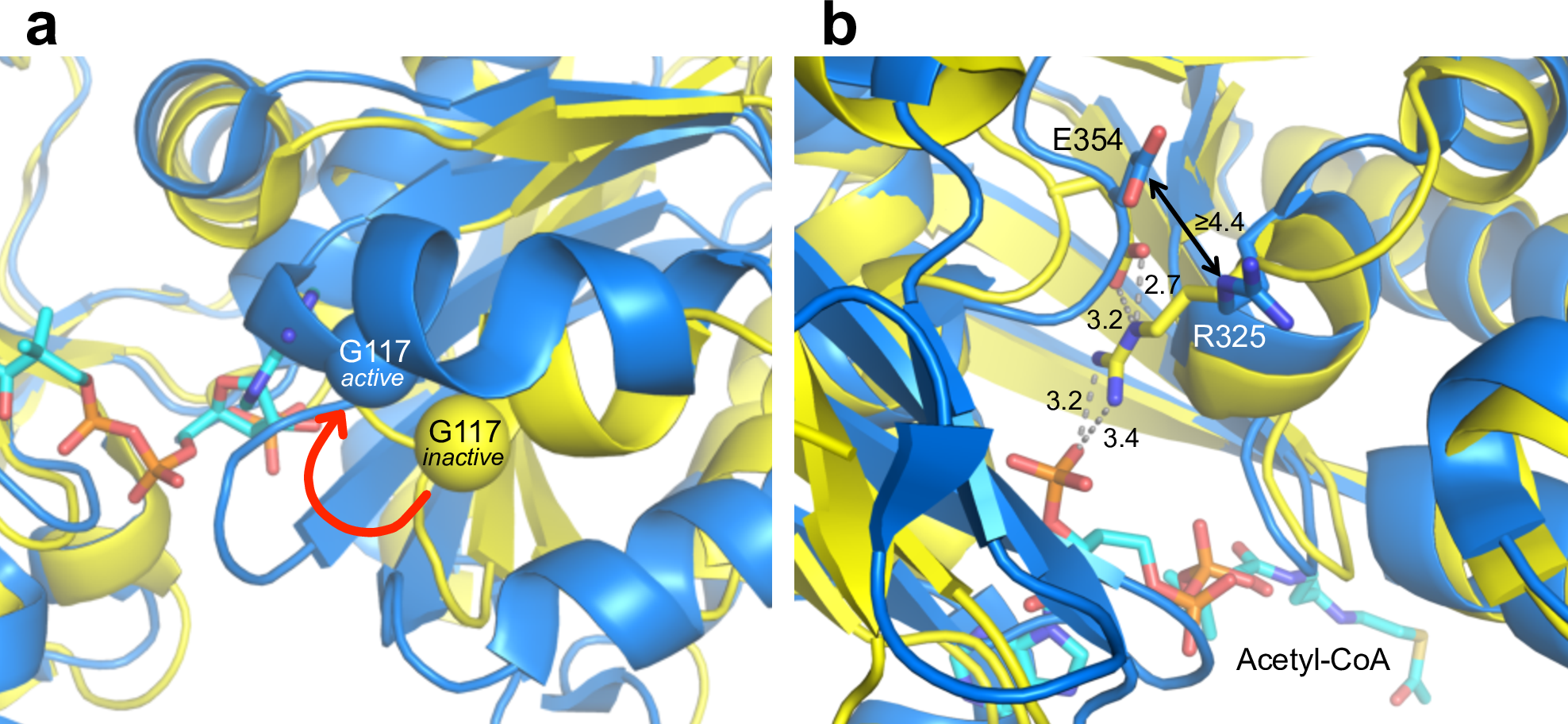

Supplement: S5 Fig — The amino acid residues of the human protein at positions 117 (indicated by the spheres in a) and 354 (side chains shown in b) correspond to the mutated residues of PfPanK1 reported in this study (at positions 95 and 507, respectively). (a) The red arrow indicates the change in the conformation of the α2-helix between the inactive and active states. The altered configuration in the active state transitions the mutated glycine that corresponds to position 117 away from the end cap of the α2-helix. (b) Dashed grey lines (inactive conformation) and the solid arrow (active conformation) represent distances between residue side chains and/or acetyl-CoA (in Å) in PanK3. A relay of interactions between Glu354, Arg325 and the 3’-phosphate of acetyl-CoA may stabilise the inactive state of the enzyme. However, in the protein’s active conformation, Glu354 and Arg325 are not within bonding distance (≥ 4.4 Å). (TIF) [file ppat.1006918.s011.tif]
